# Building a hereditary cancer program in Colombia: analysis of germline pathogenic and likely pathogenic variants spectrum in a high-risk cohort

**DOI:** 10.1038/s41431-025-01807-y

**Published:** 2025-03-10

**Authors:** María Carolina Sanabria-Salas, Ana Lucía Rivera-Herrera, María Carolina Manotas, Gonzalo Guevara, Ana Milena Gómez, Vilma Medina, Sandra Tapiero, Antonio Huertas, Marcela Nuñez, Miguel Zamir Torres, Julián Riaño-Moreno, Rafael Parra-Medina, Juan Carlos Mejía, Luis G. Carvajal-Carmona

**Affiliations:** 1 Subdirección de Investigaciones, Instituto Nacional de Cancerología, Bogotá, D.C Colombia; 2 Subdirección Médica, Instituto Nacional de Cancerología, Bogotá, D.C Colombia; 3 Division of Medical Oncology and Hematology, Princess Margaret Cancer Centre, University Health Network, Toronto, ON Canada; 4 Medical School, Universidad Cooperativa de Colombia, Villavicencio, Colombia; 5 Research Institute, Fundación Universitaria de Ciencias de la Salud, Bogotá, D.C Colombia; 6 Office of Academic Diversity, Division of Diversity, Equity and Inclusion, University of California at Davis, Davis, CA USA; 7 Department of Biochemistry and Molecular Medicine, School of Medicine, University of California Davis, Davis, CA USA; 8 Genome Center, University of California Davis, Davis, CA USA

**Keywords:** Risk factors, Genetics research

## Abstract

Genetic studies in Latin America have expanded, but further efforts are needed to understand cancer susceptibility genes beyond *BRCA1* and *BRCA2*, especially by characterizing the prevalence and spectrum of pathogenic or likely pathogenic variants (PVs) in the region. This study aimed to determine the frequency of hereditary cancer syndromes (HCS) in Colombians with solid tumors and to characterize the spectrum of PVs. Using data from the Colombia’s largest Institutional Hereditary Cancer Program, we included patients aged ≥18 years with solid tumors who met HCS criteria and were offered genetic testing with a 105-cancer gene panel. We calculated the prevalence of PVs and HCS by cancer type (beyond breast) and gene. For patients with breast cancer, we examined genotype-phenotype correlations with molecular subtypes and stratified positivity rates by different genetic testing criteria. Among 769 patients, we identified 216 PVs in 43 genes in 197 patients (26%). Thirty-three PVs were recurrent. Autosomal HCS was found in 21% (160/769) of patients (159 dominant, one recessive), while 5% (37/769) were heterozygous carriers of PVs in autosomal recessive genes. In 42% (321/769) of the cases, only one or more variants of uncertain significance (VUS) were identified, whereas 33% (251/769) had neither PVs nor VUS detected (negative results). HCS prevalence varied by cancer type (11–26%). The triple-negative subtype and bilateral presentation were strong predictors of inherited breast cancer. Our study reveals a significant presence of PVs among high-risk Colombian patients with solid tumors, underscoring the importance of genetic counseling and testing in the region.

## Introduction

Hereditary cancer syndromes (HCS) account for 5% to 10% of all malignancies worldwide and stem from germline pathogenic or likely pathogenic variants (PVs) in cancer susceptibility genes (CSGs) [1] With the increasing use of next-generation sequencing (NGS) in cancer genetics, cancer risk assessment has improved, leading to active surveillance, early cancer detection, and tailored management strategies [2]. For Latin American populations, while *BRCA1* and *BRCA2* PVs are well-known in hereditary breast and ovarian cancer syndrome (HBOC) [3–6], other HCS associated with risks for cancers beyond breast and ovarian are less well-characterized.

Disparities in clinical cancer genetics among U.S. Latinos and Latin Americans—two populations with shared socio-demographic and genetic histories—emerge from factors related to limited access to genetic services, which in turn hinder research on the spectrum of PVs in HCS within these communities [7]. This has led to gaps in human disease genetic research, which has predominantly focused on populations of European or White ancestry [8]. Colombians, the third largest Latin American population, show varied inter-regional genetic similarities with European, African, and Indigenous American populations [9–15]. Genetic studies involving patients with cancer from admixed populations are vital for enhancing our understanding of genetic diversity and its role in health disparities.

According to our current health regulations, multigene panels for germline genetic testing (GGT) are covered in Colombia for high-risk individuals meeting the National Comprehensive Cancer Network (NCCN) Guidelines [16], but many barriers to local implementation exist [2]. These barriers include limited infrastructure, training in wet-lab techniques and NGS data analysis, and insufficient education programs for healthcare professionals to identify at-risk individuals and families.

The Instituto Nacional de Cancerología in Colombia (INC-C), the largest reference cancer center in the country, delivers comprehensive, high-quality, multidisciplinary, and evidence-based care. In 2019, INC-C served 7354 new patients, decreasing to 4771 in 2020 due to COVID-19 [17, 18]. Women comprised the majority of cases both years (57.9% and 58.7%, respectively) [17, 18]. The predominant malignancies remained consistent in both periods (excluding skin cancers). For males, these were prostate, stomach, colorectum, and hematopoietic system, while for females, they were breast, cervix, thyroid, and colorectum [17, 18].

Given the significant patient volume at the INC-C, we established the largest Institutional Hereditary Cancer Program to provide comprehensive care for patients with suspected HCS, beyond Hereditary Breast and Ovarian Cancer (HBOC). Through a dedicated Registry, we aim to deepen our understanding of the molecular epidemiology of HCS, including recurrent PVs, identified in a selected cohort of high-risk Colombian patients with solid tumors.

## Materials and methods

### Type of the study and participants

This registry-based study examines the frequency and spectrum of PVs in Colombian patients with solid tumors enrolled in the Institutional Hereditary Cancer Program (referred to as the program) between April 2018 and June 2020. Details regarding the program’s conception, the founding research and clinical team, database development, implementation, and purposes are described in the Supplemental Information, Methods, and Table S1. Patients who met HCS criteria, as defined by the National Comprehensive Cancer Network (NCCN) Guidelines (2018–2020) or other international guidelines [16, 19], were admitted to the program and offered GGT. The program was open to patients from all regions of Colombia, without restrictions based on city of origin or residence. The initial database included 986 cases referred for genetic counseling (GC), comprising 540 breast cancer, 124 colorectal cancer, 68 ovarian cancer, 51 gastric cancer, 60 pediatric cancer, and 143 cases of other adult cancers. To avoid duplication of research results, exclusions were made for 12 previously published cases of pheochromocytoma and/or paraganglioma (PPGLs) [20], as well as all gastric cancer and pediatric cases that are in preparation for publication. Following these exclusions, the final dataset included 769 adults diagnosed with cancer at age ≥18 years: 491 with breast cancer, 115 with colorectal cancer, 64 with ovarian cancer, and 99 with other adult cancers, all of whom completed GGT and had available results. Signed informed consents and blood samples were collected, stored, and handled by the National Tumor Biobank Terry Fox (BNTTF, in Spanish: Banco Nacional de Tumores Terry Fox). The Scientific Committee of the INC-C approved the program as a Quality Improvement Project in 2017.

### Genetic testing

The program initially covered GC and GGT costs, which were later assumed by the Colombian Health System starting in January 2020. 105-cancer gene panel assays (customized probe panel reference # 20011891; Illumina Inc., San Diego, United States) (Supplemental Table S2), were performed in our diagnostic laboratory using a MiSeq instrument (Illumina Inc., San Diego, CA, United States) and a standardized, validated NGS method [20, 21]. Methods for germline DNA extraction, library preparation, and sequencing assays are described previously [20, 21]. Variant calling, genetic data interpretation, and methods for variant confirmation are described in Supplemental Information, Methods. Single nucleotide variants (SNVs) and small insertions and deletions (INDELs) PVs reported in this study were verified with the variant description validation software, VariantValidator [22].

### Statistical analysis

We analyzed categorical variables using frequency distributions and conducted bivariate comparisons with Fisher’s exact test or chi-square test between carriers (i.e., cases with any PVs) and non-carriers (i.e., cases with negative results, defined as those with no PVs or only variants of uncertain significance [VUS] identified by the GGT panel assay). Age at diagnosis, treated as a continuous variable, was compared between carriers and non-carriers using measures of central tendency and the two-sided Wilcoxon test. Multinomial logistic regression analyses were performed for patients with breast cancer (*n* = 491) to predict the likelihood of having a hereditary breast cancer syndrome and the likelihood of carrying a PVs in *BRCA1/2* genes (jointly and separately) or non-*BRCA* genes (dependent variables). We used patient characteristics and known GGT criteria as predictors: age at diagnosis, bilateral presentation, triple-negative breast cancer (TNBC) subtype, and positive family history (of one or more relatives diagnosed before 51 years). We calculated odds ratios (ORs), confidence intervals (CIs) at the 95% level, and *p*-values to determine the strength, precision, and significance of associations. A two-sided *p*-value < 0.05 was considered to indicate statistical significance. Statistical analyses were conducted using R Statistical Computing Software version 4.2.1 (R Foundation for Statistical Computing, Vienna, Austria).

## Results

### Patient characteristics

A total of 986 patients with cancer were referred to the program for GC from April 2018 to June 2020. Referred patients came from various regions of the country, primarily the Andean region, the Caribbean Coast, and the Western Plains. Notably, 32% were born in Bogotá, with 57.4% residing in this capital city at the time of referral (Supplemental Table S3 and Figs. S1–S2). The mean age at diagnosis among adults was under 50 years, and the majority of cases were females (84.6%; 834/986) (Supplemental Fig. S3). Over half of the patients referred for GC were diagnosed with breast cancer (55%), followed by colorectal cancer (13%) and ovarian cancer (7%) (Supplemental Fig. S3). After exclusions (see Methods section), further analyses focused exclusively on a cohort of 769 unrelated adult patients diagnosed with solid cancers at age 18 years or older who underwent GGT. In this cohort, breast cancer was the most prevalent diagnosis (64%), followed by colorectal cancer (15%), ovarian cancer (8%), and other adult cancers (13%) (Supplemental Tables S4-S5).

### Prevalence of germline sequence variations

**Fig. 1:**
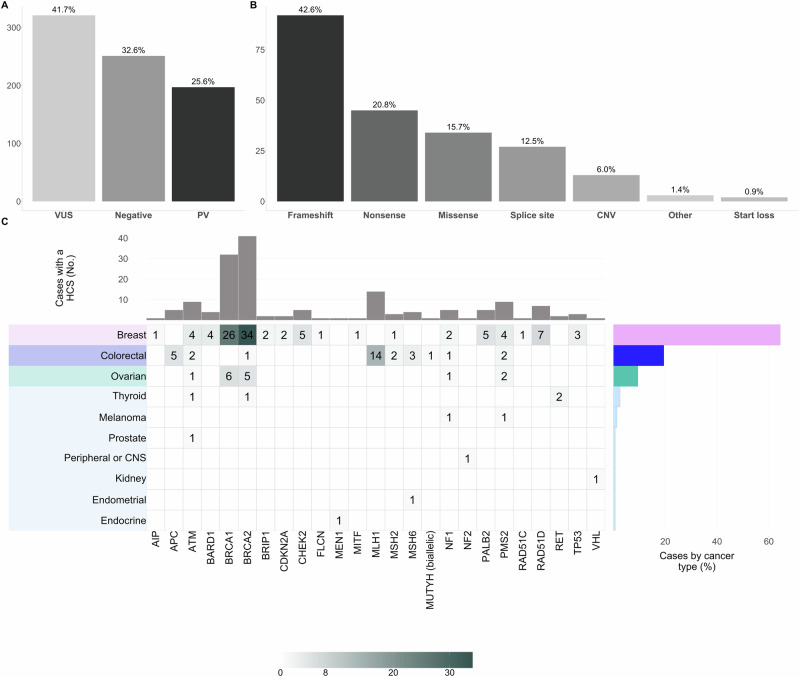
Identification, characterization and distribution of germline variants. **A** Proportion of the variant’s classification according to the ACMG guidelines and after the automated and manual curation exercise described before [21, 49]; **B** Distribution of variant type among the 216 PVs detected; **C** Heatmap of the PVs distribution by cancer type among 160 patients diagnosed with a HCS. For carriers of multiple PVs, only the affected gene with the highest cancer risk was included in the plot. Each heatmap cell shows the color-gradient indicating the range (as specified in the color-key legend at the bottom) and absolute number of patients with a specific type of cancer (rows; y-axis) that are carriers of a heterozygous germline PV on a specific gene (columns; x-axis), except for one patient with colorectal cancer and biallelic *MUTYH* PVs (compound heterozygous). The later corresponds to an autosomal recessive syndrome, while all the rest were diagnosed with an autosomal dominant condition. The right-hand bar plot displays the relative frequency (%) of cases diagnosed with a HCS by cancer type. The upper bar plot shows the absolute frequency (No.) of PVs per gene. The type of cancer is color-coded. ACMG, The American College of Medical Genetics and Genomics; PVs, Pathogenic/Likely Pathogenic variants; HCS, Hereditary Cancer Syndrome.

Overall, we found 216 PVs in 26% (197/769) of the patients (Table 1, Fig. 1A, and Supplemental Tables S4-S5). Among the 197 carriers, 18 had more than one PV, while 179 had only one (Table 1 and Table 2). In 42% (321/769) of the cases, one or more VUS were identified, whereas 33% (251/769) showed no PVs or VUS detected by the 105-gene panel assay used for GGT (i.e., negative result) (Fig. 1A, and Supplemental Tables S4-S5). Interestingly, 25% of the 197 cases with PVs also had one or more VUS (data not shown), although further analyses focused on the most clinically relevant genetic findings.

**Table 1:** Description of PVs in cancer genes found among 197 Colombians adults with solid cancers/tumors.

**Gene**	**OMIM code / Phenotypic Series Code**	**Associated Disease Names**	**Genetic Variant**	**Molecular Consequence**	**N° of carriers**	**Cancer Type (n)**	**Age at diagnosis (years)**
*AIP (NM_003977.4)*	219090, 102200, 600634	PITUITARY ADENOMA, ACTH-SECRETING, PITUITARY ADENOMA, GROWTH HORMONE-SECRETING, 1, PITUITARY ADENOMA, PROLACTIN-SECRETING	*AIP:c.619del p.(Ala207ProfsTer8)*	Frameshift	1	Breast	66
*APC (NM_000038.6)*	114550, PS175100, 155255, 135290, 114500, 613659	DESMOID DISEASE, HEREDITARY, HEPATOCELLULAR CARCINOMA, FAMILIAL ADENOMATOUS POLYPOSIS, COLORECTAL CANCER, MEDULLOBLASTOMA, GASTRIC CANCER	*APC:c.1263* *G* > *A p.(Trp421Ter)*	Nonsense	1	Colorectal	67
			*APC:c.1548+1* *G* > *C p.?*	Intron variant	1	Colorectal	35
			*APC:c.4366_4369del p.(Lys1456HisfsTer16)*	Frameshift	1	Colorectal	41
			*APC:c.532-8* *G* > *A p.?*	Intron variant	1	Colorectal	31
			*APC: whole-gene deletion*	Indels - CNV	1	Colorectal	27
*ATM (NM_000051.4)*	208900, 114480	BREAST CANCER, ATAXIA-TELANGIECTASIA	*ATM:c.3510dup p.(Gln1171ThrfsTer8)*	Frameshift	1	Breast	46
			*ATM:c.7767del p.(Lys2589AsnfsTer17)*	Frameshift	1	Breast ^a^	47
			*ATM:c.8264_8268del p.(Tyr2755CysfsTer12)*	Frameshift	1	Breast	38
			*ATM:c.8419-2* *A* > *C p.?*	Intron variant	1	Breast	34
			*ATM:c.9139* *C* > *T p.(Arg3047Ter)*	Nonsense	1	Thyroid	35
			***ATM: deletion exons 27-29***	**Indels - CNV**	**4**	Colorectal (2)	54 ^b^
						Ovarian (1)	52
						Prostate (1)	82
*BARD1 (NM_000465.4)*	114480	BREAST CANCER	***BARD1:c.2229dup p.(Asn744Ter)***	**Nonsense**	**4**	Breast (4) ^a^	44.5 ^b^
*BLM (NM_000057.4)*	210900	BLOOM SYNDROME	*BLM:c.3415* *C* > *T p.(Arg1139Ter)*	Nonsense	1	Breast ^a^	48
*BRCA1 (NM_007294.4)*	PS227650, 167000, 614320, PS604370, 114480	OVARIAN CANCER, PANCREATIC CANCER, SUSCEPTIBILITY TO, 4, BREAST CANCER, BREAST-OVARIAN CANCER, FAMILIAL, SUSCEPTIBILITY TO, FANCONI ANEMIA	***BRCA1:c.1674del p.(Gly559ValfsTer13)***	**Frameshift**	**8**	Breast (6) ^a^	55.5 ^b^
						Ovarian (2)	44.5 ^b^
			*BRCA1:c.1918C>T p.(Gln640Ter)*	Nonsense	1	Breast	44
			*BRCA1:c.212+1* *G* > *T p.?*	Intron variant	1	Breast	49
			*BRCA1:c.213-12* *A* > *G p.?*	Intron variant	1	Breast	69
			*BRCA1:c.2801del p.(Gln934ArgfsTer66)*	Frameshift	1	Breast	36
			*BRCA1:c.3270del p.(Gln1090HisfsTer19)*	Frameshift	1	Breast	47
			***BRCA1:c.3331_3334del p.(Gln1111AsnfsTer5)***	**Frameshift**	**6**	Breast (5) ^a^	37 ^b^
						Ovarian (1)	51
			*BRCA1:c.3616del p.(Ala1206ProfsTer4)*	Frameshift	1	Breast	28
			*BRCA1:c.3679* *C* > *T p.(Gln1227Ter)*	Nonsense	1	Breast	37
			*BRCA1:c.4503* *C* > *A p.(Cys1501Ter)*	Nonsense	1	Breast	41
			*BRCA1:c.5090* *G* > *A p.(Cys1697Tyr)*	Missense	1	Breast ^a^	35
			***BRCA1:c.5123*** ***C*** > ***A p.(Ala1708Glu)***	**Missense**	**8**	Breast (5)	35 ^b^
						Ovarian (3) ^a^	56 ^b^
			*BRCA1:c.5365* *G* > *A p.(Ala1789Thr)*	Missense	1	Breast	33
			*BRCA1:c.5560del p.(Leu1854Ter)*	Nonsense	1	Breast	35
*BRCA2 (NM_000059.4)*	PS227650, PS194070, 155255, 11448, 176807, PS137800, 613347, PS604370	BREAST-OVARIAN CANCER, FAMILIAL, SUSCEPTIBILITY TO, BREAST CANCER, MEDULLOBLASTOMA, PROSTATE CANCER, PANCREATIC CANCER, SUSCEPTIBILITY TO, 2, GLIOMA, WILMS TUMOR, FANCONI ANEMIA	***BRCA2:c.1763_1766del p.(Asn588SerfsTer25)***	**Frameshift**	**6**	Breast (5)	41 ^b^
						Ovarian (1)	50
			*BRCA2:c.1947del p.(Asn650MetfsTer10)*	Frameshift	1	Ovarian	50
			***BRCA2:c.2808_2811del p.(Ala938ProfsTer21)***	**Frameshift**	**7**	Breast (7) ^a^	39 ^b^
			***BRCA2:c.3860del p.(Asn1287IlefsTer6)***	**Frameshift**	**2**	Breast (2)	59 ^b^
			*BRCA2:c.4435_4439del p.(Ser1479Ter)*	Nonsense	1	Ovarian	49
			***BRCA2:c.4889*** ***C*** > ***G p.(Ser1630Ter)***	**Nonsense**	**3**	Breast (2)	48 ^b^
						Ovarian (1)	56
			*BRCA2:c.5217_5220del p.(Tyr1739Ter)*	Nonsense	1	Breast	44
			*BRCA2:c.5720_5723del p.(Ser1907Ter)*	Nonsense	1	Colorectal	56
			*BRCA2:c.5851_5854del p.(Ser1951TrpfsTer11)*	Frameshift	1	Breast	60
			*BRCA2:c.6014del p.(Asp2005ValfsTer35)*	Frameshift	1	Breast	56
			***BRCA2:c.6024dup p.(Gln2009AlafsTer9)***	**Frameshift**	**4**	Breast (3)	42 ^b^
						Ovarian (1) ^a^	48
			***BRCA2:c.6082_6083del p.(Glu2028ArgfsTer20)***	**Frameshift**	**2**	Breast (2)	45 ^b^
			***BRCA2:c.6275_6276del p.(Leu2092ProfsTer7)***	**Frameshift**	**2**	Breast (2)	52.5 ^b^
			***BRCA2:c.632-3*** ***C*** > ***G p.?***	**Intron variant**	**2**	Breast (2) ^a^	69 ^b^
			*BRCA2:c.6884_6888del p.(Arg2295AsnfsTer43)*	Frameshift	1	Breast	56
			*BRCA2:c.7558* *C* > *T p.(Arg2520Ter)*	Nonsense	1	Breast ^a^	53
			***BRCA2: deletion exons 1-15***	**Indels - CNV**	**5**	Breast (4)	52 ^b^
						Ovarian (1)	55
			*BRCA2: deletion exons 12-17*	Indels - CNV	1	Thyroid	39
*BRIP1 (NM_032043.3)*	PS227650, 114480	BREAST CANCER, FANCONI ANEMIA	*BRIP1:c.2714del p.(Asn905MetfsTer7)*	Frameshift	1	Breast	65
			*BRIP1:c.2992_2993del p.(Lys998GlufsTer3)*	Frameshift	1	Breast	65
*BUB1B (NM_001211.6)*	PS257300, 176430, 114500	COLORECTAL CANCER, PREMATURE CHROMATID SEPARATION TRAIT, MOSAIC VARIEGATED ANEUPLOIDY SYNDROME	***BUB1B:c.199*** ***C*** > ***T p.(Arg67Ter)***	**Nonsense**	**3**	Breast (2) ^a^	47.5 ^b^
						Ovarian (1)	50
			*BUB1B:c.638del p.(Glu213GlyfsTer15)*	Frameshift	1	Lung	57
*CDKN2A (NM_058195.4;*	PS151623, PS155600, 606719, 155755	MELANOMA-ASTROCYTOMA SYNDROME, MELANOMA-PANCREATIC CANCER SYNDROME, LI-FRAUMENI SYNDROME, MELANOMA, CUTANEOUS MALIGNANT	***CDKN2A:c.194-3653*** ***G*** > ***T p.?***	**5 UTR**	**2**	Breast (2)	51.5 ^b^
*NM_000077.5)*			*CDKN2A:c.458-105* *A* > *G p.?*	Intron variant	1	Breast ^a^	34
*CEP57 (NM_014679.5)*	PS257300	MOSAIC VARIEGATED ANEUPLOIDY SYNDROME	*CEP57:c.1388_1391del p.(Lys463IlefsTer3)*	Frameshift	1	Kidney ^a^	44
			*CEP57:c.915_925dup p.(Leu309ProfsTer9)*	Frameshift	1	Breast	56
*CHEK2 (NM_007194.4)*	PS151623, 176807, 259500, 114480	LI-FRAUMENI SYNDROME, BREAST CANCER, PROSTATE CANCER, OSTEOGENIC SARCOMA	*CHEK2:c.1427* *C* > *T p.(Thr476Met)*	Missense	1	Breast	36
			*CHEK2:c.349* *A* > *G p.(Arg117Gly)*	Missense	1	Breast	55
			*CHEK2:c.591del p.(Val198PhefsTer7)*	Frameshift	1	Breast	43
			*CHEK2:c.846+1* *G* > *C p.?*	Intron variant	1	Breast	56
			*CHEK2: deletion exons 8-12*	Indels - CNV	1	Breast	38
*ERCC4 (NM_005236.3)*	278760, PS227650, 610965	XERODERMA PIGMENTOSUM, COMPLEMENTATION GROUP F, XFE PROGEROID SYNDROME, FANCONI ANEMIA	*ERCC4:c.22* *C* > *T p.(Arg8Ter)*	Nonsense	1	Thyroid	62
*ERCC5 (NM_000123.4)*	PS214150, 278780	CEREBROOCULOFACIOSKELETAL SYNDROME, XERODERMA PIGMENTOSUM, COMPLEMENTATION GROUP G	*ERCC5:c.2929_2930del p.(Leu977ValfsTer17)*	Frameshift	1	Breast	37
*FANCA (NM_000135.4)*	PS227650	FANCONI ANEMIA	*FANCA:c.2641* *C* > *T p.(Gln881Ter)*	Nonsense	1	Colorectal ^a^	59
			*FANCA:c.2778+1* *G* > *A p.?*	Intron variant	1	Breast	44
			*FANCA:c.718* *C* > *T p.(Gln240Ter)*	Nonsense	1	Breast	34
*FANCD2 (NM_001018115.3)*	PS227650	FANCONI ANEMIA	***FANCD2:c.2444*** ***G*** > ***A p.(Arg815Gln)***	**Missense**	**2**	Breast (1) ^a^	53
						Pancreas (1)	28
*FANCL (NM_018062.4)*	PS227650	FANCONI ANEMIA	***FANCL:c.1051_1052del p.(Ser351PhefsTer2)***	**Frameshift**	**2**	Breast (1)	50
						Colorectal (1)	58
*FANCM (NM_020937.4)*	PS227650	FANCONI ANEMIA	*FANCM:c.4054* *A* > *T p.(Lys1352Ter)*	Nonsense	1	Breast	37
*FLCN (NM_144997.7)*	135150, 144700, 173600, 114500	RENAL CELL CARCINOMA, NONPAPILLARY, PNEUMOTHORAX, PRIMARY SPONTANEOUS, BIRT-HOGG-DUBE SYNDROME, COLORECTAL CANCER	***FLCN:c.1285del p.(His429ThrfsTer39)***	**Frameshift**	**2**	Breast (1) ^a^	53
						Colorectal (1) ^a^	48
*MEN1 (NM_001370259.2)*	145000, PS131100	HYPERPARATHYROIDISM 1, MULTIPLE ENDOCRINE NEOPLASIA	*MEN1:c.1660* *C* > *T p.(Gln554Ter)*	Nonsense	1	Pituitary Adenoma	21
*MITF (NM_001354604.2)*	103500, PS155600, PS193500	TIETZ SYNDROME, MELANOMA, CUTANEOUS MALIGNANT, WAARDENBURG SYNDROME	*MITF:c.1273* *G* > *A p.(Glu425Lys)*	Missense	1	Breast	42
*MLH1 (NM_000249.4)*	276300, 158320, PS120435	MUIR-TORRE SYNDROME, MISMATCH REPAIR CANCER SYNDROME, COLORECTAL CANCER, HEREDITARY NONPOLYPOSIS	*MLH1:c.1038* *G* > *A p.(Gln346* = *)*	Synonymous	1	Colorectal	25
			***MLH1:c.1852_1854del p.(Lys618del)***	**Indel - Inframe**	**2**	Colorectal (2)	44 ^b^
			***MLH1:c.1918C>T p.(Pro640Ser)***	**Missense**	**4**	Colorectal (4) ^a^	55.5 ^b^
			*MLH1:c.209_215del p.(Lys70IlefsTer20)*	Frameshift	1	Colorectal	56
			*MLH1:c.676* *C* > *T p.(Arg226Ter)*	Nonsense	1	Colorectal	38
			***MLH1:c.790+1*** ***G*** > ***A p.?***	**Intron variant**	**5**	Colorectal (5)	57 ^b^
*MSH2 (NM_000251.3)*	276300, 158320, PS120435	MUIR-TORRE SYNDROME, MISMATCH REPAIR CANCER SYNDROME, COLORECTAL CANCER, HEREDITARY NONPOLYPOSIS	*MSH2:c.1276* *G* > *A p.(Gly426Arg)*	Missense	1	Colorectal	59
			*MSH2:c.528_529del p.(Cys176Ter)*	Nonsense	1	Colorectal	41
			*MSH2:c.942+3* *A* > *T p.?*	Intron variant	1	Breast	56
*MSH6 (NM_000179.3)*	276300, PS120435, 608089	ENDOMETRIAL CANCER, MISMATCH REPAIR CANCER SYNDROME, COLORECTAL CANCER, HEREDITARY NONPOLYPOSIS	*MSH6:c.1994_1995del p.(Glu665ValfsTer2)*	Frameshift	1	Endometrial	56
			*MSH6:c.3766dup p.(Tyr1256LeufsTer19)*	Frameshift	1	Breast ^a^	37
			*MSH6:c.3794del p.(Gly1265AspfsTer7)*	Frameshift	1	Colorectal	56
			***MSH6:c.742del p.(Arg248GlufsTer31)***	**Frameshift**	**2**	Colorectal (2)	60.5 ^b^
*MUTYH (NM_001048174.2)*	132600, 613659, PS175100	PILOMATRIXOMA, FAMILIAL ADENOMATOUS POLYPOSIS, GASTRIC CANCER	***MUTYH:c.1143_1144dup p.(Glu382GlyfsTer43)***	**Frameshift**	**2**	Colorectal (1)	71
						Kidney (1) ^a^	44
			***MUTYH:c.1103*** ***G*** > ***A p.(Gly368Asp)***	**Missense**	**7**	Breast (5) ^a^	59 ^b^
						Ovarian (1)	32
						Colorectal (1) ^a^	54
			*MUTYH:c.452* *A* > *G p.(Tyr151Cys)*	Missense	1	Colorectal (1) ^a^	54
			*MUTYH:c.604* *C* > *T p.(Gln202Ter)*	Nonsense	1	Breast ^a^	48
*NBN (NM_002485.5)*	609135, 613065, 114480, 251260	BREAST CANCER, LEUKEMIA, ACUTE LYMPHOBLASTIC, APLASTIC ANEMIA, NIJMEGEN BREAKAGE SYNDROME	***NBN:c.1515del p.(Glu505AspfsTer24)***	**Frameshift**	**2**	Breast (1)	39
						Colorectal (1)	63
*NF1 (NM_001042492.3)*	607785, 193520, 162200, 162210, 601321	NEUROFIBROMATOSIS, TYPE I, JUVENILE MYELOMONOCYTIC LEUKEMIA, NEUROFIBROMATOSIS-NOONAN SYNDROME, NEUROFIBROMATOSIS, FAMILIAL SPINAL, WATSON SYNDROME	*NF1:c.3721* *C* > *T p.(Arg1241Ter)*	Nonsense	1	Breast	53
			*NF1:c.4245* *T* > *A p.(Asn1415Lys)*	Missense	1	Breast	48
			*NF1:c.574* *C* > *T p.(Arg192Ter)*	Nonsense	1	Colorectal ^a^	59
			*NF1:c.706* *C* > *T p.(Gln236Ter)*	Nonsense	1	Ovarian	52
			*NF1:c.7909* *C* > *T p.(Arg2637Ter)*	Nonsense	1	Melanoma	21
*NF2 (NM_000268.4)*	607174, 101000, PS162091	SCHWANNOMATOSIS, NEUROFIBROMATOSIS, TYPE II, MENINGIOMA, FAMILIAL, SUSCEPTIBILITY TO	*NF2:c.676-2* *A* > *C p.?*	Intron variant	1	Peripheral or CNS	33
*NTHL1 (NM_002528.7)*	PS175100	FAMILIAL ADENOMATOUS POLYPOSIS	*NTHL1:c.244* *C* > *T p.(Gln82Ter)*	Nonsense	1	Breast	52
*PALB2 (NM_024675.4)*	PS227650, 114480, 613348	BREAST CANCER, PANCREATIC CANCER, SUSCEPTIBILITY TO, 3, FANCONI ANEMIA	***PALB2:c.2288_2291del p.(His762_Leu763insTer)***	**Nonsense**	**4**	Breast (4)	50.5 ^b^
			*PALB2:c.3256* *C* > *T p.(Arg1086Ter)*	Nonsense	1	Breast	55
*PMS2 (NM_000535.7)*	276300, PS120435	MISMATCH REPAIR CANCER SYNDROME, COLORECTAL CANCER, HEREDITARY NONPOLYPOSIS	*PMS2:c.1855del p.(Asp619ThrfsTer4)*	Frameshift	1	Breast ^a^	58
			***PMS2:c.2182_2184delinsG p.(Thr728AlafsTer7)***	**Frameshift**	**6**	Breast (3)	41 ^b^
						Melanoma (1)	23
						Ovarian (2)	54.5 ^b^
			*PMS2:c.354-1* *G* > *A p.?*	Intron variant	1	Breast	55
			*PMS2:c.400* *C* > *T p.(Arg134Ter)*	Nonsense	1	Colorectal	55
			*PMS2: whole-gene deletion*	Indels - CNV	1	Colorectal	34
*RAD50 (NM_005732.4)*	613078	NIJMEGEN BREAKAGE SYNDROME-LIKE DISORDER	***RAD50:c.2165dup p.(Glu723GlyfsTer5)***	**Frameshift**	**2**	Breast (2)	47 ^b^
			*RAD50:c.3598* *C* > *T p.(Arg1200Ter)*	Nonsense	1	Breast	49
*RAD51C (NM_058216.3)*	PS227650, PS604370	BREAST-OVARIAN CANCER, FAMILIAL, SUSCEPTIBILITY TO, FANCONI ANEMIA	*RAD51C:c.561_562del p.(His187GlnfsTer15)*	Frameshift	1	Breast	45
*RAD51D (NM_002878.4)*	PS604370	BREAST-OVARIAN CANCER, FAMILIAL, SUSCEPTIBILITY TO	***RAD51D:c.556*** ***C*** > ***T p.(Arg186Ter)***	**Nonsense**	**3**	Breast (3) ^a^	42 ^b^
			***RAD51D:c.94_95del p.(Val32PhefsTer38)***	**Frameshift**	**5**	Breast (5)	45 ^b^
*RECQL4 (NM_004260.4)*	268400, 218600, 266280	RAPADILINO SYNDROME, ROTHMUND-THOMSON SYNDROME, BALLER-GEROLD SYNDROME	***RECQL4:c.1048_1049del p.(Arg350GlyfsTer21)***	**Frameshift**	**3**	Breast (2) ^a^	52 ^b^
						Colorectal (1)	18
*RET (NM_020975.6)*	171300, 155240, PS142623, 209880, 191830, PS131100	HIRSCHSPRUNG DISEASE, MULTIPLE ENDOCRINE NEOPLASIA, THYROID CARCINOMA, FAMILIAL MEDULLARY, CENTRAL HYPOVENTILATION SYNDROME, CONGENITAL, PHEOCHROMOCYTOMA, RENAL HYPODYSPLASIA/APLASIA 1	*RET:c.1900T>C p.(Cys634Arg)*	Missense	1	Thyroid	47
			*RET:c.2752* *A* > *T p.(Met918Leu)*	Missense	1	Thyroid	42
*SBDS (NM_016038.4)*	260400, 609135	SHWACHMAN-DIAMOND SYNDROME, APLASTIC ANEMIA	***SBDS:c.258+2*** ***T*** > ***C p.?***	**Intron variant**	**8**	Breast (6) ^a^	51 ^b^
						Colorectal (1)	62
						Pancreas (1)	35
*SDHA (NM_004168.4)*	252011, 604287, PS115200, PS168000, 256000	LEIGH SYNDROME, DILATED CARDIOMYOPATHY, CARNEY TRIAD, PARAGANGLIOMAS, MITOCHONDRIAL COMPLEX II DEFICIENCY	*SDHA:c.667del p.(Asp223IlefsTer3)*	Frameshift	1	Breast	40
*SLX4 (NM_032444.4)*	PS227650	FANCONI ANEMIA	*SLX4:c.3895_3896del p.(Arg1299GlyfsTer35)*	Frameshift	1	Colorectal	61
			*SLX4:c.838* *G* > *T p.(Gly280Ter)*	Nonsense	1	Colorectal	42
*TP53 (NM_000546.6)*	114550, 260500, 605027, PS137800, 614740, 114480, 260350, 607107, 202300, 114500, PS151623, 259500	ADRENOCORTICAL CARCINOMA, HEREDITARY, PANCREATIC CANCER, BASAL CELL CARCINOMA, SUSCEPTIBILITY TO, 7, HEPATOCELLULAR CARCINOMA, PAPILLOMA OF CHOROID PLEXUS, LI-FRAUMENI SYNDROME, NASOPHARYNGEAL CARCINOMA, OSTEOGENIC SARCOMA, COLORECTAL CANCER, BREAST CANCER, LYMPHOMA, NON-HODGKIN, FAMILIAL, GLIOMA	*TP53:c.374* *C* > *T p.(Thr125Met)*	Missense	1	Breast	51
			*TP53:c.473* *G* > *T p.(Arg158Leu)*	Missense	1	Breast	37
			*TP53:c.818* *G* > *A p.(Arg273His)*	Missense	1	Breast ^a^	37
*VHL (NM_000551.4)*	193300, 144700, PS133100, 171300	RENAL CELL CARCINOMA, NONPAPILLARY, ERYTHROCYTOSIS, FAMILIAL, VON	*VHL:c.549_550del p.(Tyr185ArgfsTer70)*	Frameshift	1	Kidney	35
		HIPPEL-LINDAU SYNDROME, PHEOCHROMOCYTOMA					
*WRN (NM_000553.6)*	277700	WERNER SYNDROME	*WRN:c.504+1* *G* > *A p.?*	Intron variant	1	Breast	53
*XPC (NM_004628.5)*	278720, 278700	XERODERMA PIGMENTOSUM, COMPLEMENTATION GROUP A, XERODERMA	*XPC:c.1103_1104del p.(Gln368ArgfsTer6)*	Frameshift	1	Breast	52
		PIGMENTOSUM, COMPLEMENTATION GROUP C					

*PVs* Pathogenic/Likely Pathogenic variants.

^a^Counts include carriers of two or more PVs.

^b^Median.

Bold PVs indicate that these were found in two unrelated patients or more (recurrent PVs).

**Table 2:** Carriers of more than one pathogenic/likely pathogenic variants (PVs).

Cancer	Genes^a^ (Age at diagnosis)	Genetic Variants (PVs)	HCS
Type			
Breast	*BRCA2 - CDKN2A* (34)	***BRCA2:c.2808_2811del p.(Ala938ProfsTer21)***	HBOC & FAMMMPC
		***CDKN2A:c.458-105*** ***A*** > ***G p.?***	
	*BRCA1 - BRCA1* (35)	***BRCA1:c.1674del p.(Gly559ValfsTer13)***	HBOC
		***BRCA1:c.5090*** ***G*** > ***A p.(Cys1697Tyr)***	
	*TP53 - MUTYH* (37)	***TP53:c.818*** ***G*** > ***A p.(Arg273His)***	LF
		*MUTYH:c.1103* *G* > *A p.(Gly368Asp)*	
	*BRCA1 - MSH6* (37)	***BRCA1:c.3331_3334del p.(Gln1111AsnfsTer5)***	HBOC & LS
		***MSH6:c.3766dup p.(Tyr1256LeufsTer19)***	
	*ATM - RAD51D* (47)	***ATM:c.7767del p.(Lys2589AsnfsTer17)***	HBOC (Non-BRCA)
		***RAD51D:c.556*** ***C*** > ***T p.(Arg186Ter)***	
	*MUTYH - BLM* (48)	*MUTYH:c.604* *C* > *T p.(Gln202Ter)*	NO
		*BLM:c.3415* *C* > *T p.(Arg1139Ter)*	
	*BRCA2 - FANCD2 - SBDS* (53)	***BRCA2:c.7558*** ***C*** > ***T p.(Arg2520Ter)***	HBOC
		*FANCD2:c.2444* *G* > *A p.(Arg815Gln)*	
		*SBDS:c.258+2* *T* > *C p.?*	
	*FLCN - SBDS* (53)	***FLCN:c.1285del p.(His429ThrfsTer39)***	BHD
		*SBDS:c.258+2* *T* > *C p.?*	
	*BRCA1 - PMS2* (58)	***BRCA1:c.1674del p.(Gly559ValfsTer13)***	HBOC & LS
		***PMS2:c.1855del p.(Asp619ThrfsTer4)***	
	*BARD1 - MUTYH* (59)	***BARD1:c.2229dup p.(Asn744Ter)***	HBOC (Non-BRCA)
		*MUTYH:c.1103* *G* > *A p.(Gly368Asp)*	
	*SBDS - BUB1B* (62)	*SBDS:c.258+2* *T* > *C p.?*	NO
		*BUB1B:c.199* *C* > *T p.(Arg67Ter)*	
	*MUTYH - RECQL4* (65)	*MUTYH:c.1103* *G* > *A p.(Gly368Asp)*	NO
		*RECQL4:c.1048_1049del p.(Arg350GlyfsTer21)*	
	*BRCA2 - MUTYH* (85)	***BRCA2:c.632-3*** ***C*** > ***G p.?***	HBOC
		*MUTYH:c.1103* *G* > *A p.(Gly368Asp)*	
Colorecta**l**	*MLH1 - FLCN* (48)	***MLH1:c.1918C>T p.(Pro640Ser)***	LS & BHD
		***FLCN:c.1285del p.(His429ThrfsTer39)***	
	*MUTYH - MUTYH* (54)^b^	***MUTYH:c.1103*** ***G*** > ***A p.(Gly368Asp)***	MAP
		***MUTYH:c.452*** ***A*** > ***G p.(Tyr151Cys)***	
	*NF1 - FANCA* (59)	***NF1:c.574*** ***C*** > ***T p.(Arg192Ter)***	NF1
		*FANCA:c.2641* *C* > *T p.(Gln881Ter)*	
Ovarian	*BRCA1 - BRCA2* (48)	***BRCA1:c.5123*** ***C*** > ***A p.(Ala1708Glu)***	HBOC
		***BRCA2:c.6024dup p.(Gln2009AlafsTer9)***	
Kidney	*MUTYH - CEP57* (44)	*MUTYH:c.1143_1144dup p.(Glu382GlyfsTer43)*	NO
		*CEP57:c.1388_1391del p.(Lys463IlefsTer3)*	

*PVs* Pathogenic/Likely Pathogenic variants, *HCS* Hereditary Cancer Syndrome, *HBOC* hereditary breast and ovarian cancer, *LS* Lynch syndrome, *NF1* neurofibromatosis type 1, *LF* Li-Fraumeni syndrome, *FAMMMPC* familial atypical multiple mole melanoma-pancreatic carcinoma syndrome, *BHD* Birt-Hogg Dube syndrome, *MAP* MUTYH-associated polyposis.

^a^Double heterozygous PVs found in patients with cancer registered in the Institutional Hereditary Cancer Program (except for one patient with colorectal cancer^b^).

^b^Compound heterozygous in a patient with colorectal cancer diagnosed with MAP of autosomal recessive inheritance.

Bold PVs indicate their association with a HCS.

The 216 PVs were distributed in 43 genes from the panel and 120/216 were unique. The rest corresponded to any of the 33 recurrent PVs in 19 genes in our Colombian cohort (Table 1). Most PVs were loss-of-function (63.4%; 92 frameshift and 45 nonsense) and copy number variants (CNV) accounted for a 6% (*n* = 13) (Fig. 1B).

Of the cases analyzed, 21% (160/769) were diagnosed with a HCS based on GGT results. This group includes one patient with colorectal cancer and five with breast cancer, where the identified genetic alteration is not typically associated with their cancer type, referred to as “incidental cases” (Table 1, Fig. 1C, and Supplemental Tables S4-S5). Additionally, 5% (37/769) of cases had heterozygous PVs in genes that are exclusively associated with a recessive inheritance pattern, such as *NTHL1, FANCM*, and *MUTYH*, among others (Table 1 and Supplemental Tables S4-S5).

### Clinical characteristics in carriers of PVs compared to non-carriers

Clinical and demographic characteristics of patients diagnosed with breast, colorectal or ovarian cancer are summarized in Supplemental Table S4. Carriers were defined as patients with any PV in any gene (i.e., HCS cases and carriers of a recessive condition). Non-carriers were those with negative GGT results, indicating no PVs or VUS were identified by the 105-cancer gene panel.

Among 491 patients with breast cancer, 26% had PVs, 41% had only one or more VUS, and 33% were non-carriers (Supplemental Table S4). Ductal breast cancer was the most frequent (88%), and in the carrier group, most cancers were poorly differentiated compared to the non-carrier group (*p*-value < 0.01).

For cases with cancers different than breast, the percentage of those with PVs, VUS and non-carriers, were: 33%, 48% and 19% in 115 colorectal cancer cases; 26%, 38% and 36% in 64 ovarian cancer cases; and 16%, 41% and 42% in 99 cases within the other adult cancer category (Supplemental Tables S4-S5). No significant differences were observed between carriers and non-carrier cases affected by these cancer types, although, more cases with absent staining for one or more mismatch repair (MMR) proteins were identified in the carriers group affected by colorectal cancer compared to non-carriers (*p*-value 0.07). All cases with MSH6 loss had a PV in that gene, while over half of cases with MLH1/PMS2 loss had a PV in *MLH1* PVs (Supplemental Fig. S4).

### HCS frequency by cancer type

Figure 1C depicts PVs per gene among 160 of 769 (21%) patients diagnosed with HCS. HBOC was the most frequent with 9% (73/769) due to PVs in *BRCA2* (*n* = 41) or *BRCA1* (*n* = 32) genes. Other homologous recombination DNA damage repair (HR-DDR) genes associated with other cancer risks besides breast and ovarian, such as *ATM* (*n* = 9), *BARD1* (*n* = 4), *BRIP1* (*n* = 2), *CHEK2* (*n* = 5), *PALB2* (*n* = 5), *RAD51C* (*n* = 1), and *RAD51D* (*n* = 7), accounted for an additional 4% (33/769). Lynch syndrome (LS) was the second most frequently diagnosed, with a prevalence of 4% (30/769). PVs in *MLH1* (*n* = 14) and *PMS2* (*n* = 9) were the most common, followed by *MSH6* (*n* = 4) and *MSH2* (*n* = 3). Familial adenomatous polyposis (FAP), neurofibromatosis type 1 (NF1), and Li-Fraumeni (LF) followed, with an estimated prevalence of 0.65% [5 *APC*, 5 *NF1*], and 0.39% [3 *TP53*]. Other less frequent HCS are listed in Supplemental Fig. S5.

**Fig. 2:**
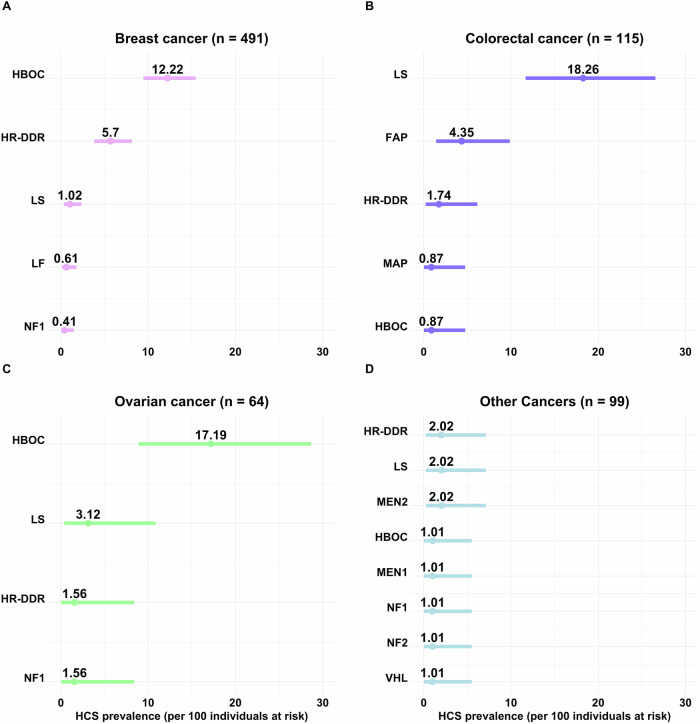
Prevalence of HCS calculated for adult cancer cases at risk by cancer type. Ranked error bar plot of the prevalence calculation for each HCS, and its 95% confidence interval, per 100 adult cancer cases at risk (received GC and GGT for suspicious of an inherited condition), are shown for **A**. Breast (*n* = 491); **B**. Colorectal (*n* = 115); **C** Ovarian (*n* = 64); and **D**. Other cancers in adults (*n* = 99). For carriers of multiple PVs, only the HCS (i.e., gene) with the highest cancer risk known to be associated with the patient’s cancer type was included in the plot. Incidental findings due to mutated genes not accepted to be associated with the patient’s cancer type, were excluded. HBOC, hereditary breast and ovarian cancer *BRCA1* and *BRCA2* related; HR-DDR, homologous recombination DNA damage repair (i.e., *ATM*, *BARD1*, *BRIP1*, *CHEK2*, *PALB2*, *RAD51C*, and *RAD51D*) associated cancer risk; LS, Lynch syndrome (i.e, *MLH1*, *PMS2*, *MSH6* and *MSH2*); FAP, familial adenomatous polyposis (i.e., *APC*); NF1, neurofibromatosis type 1 (i.e., *NF1*); LF, Li-Fraumeni syndrome (i.e., *TP53*); MEN2, multiple endocrine neoplasia type 2 (i.e., *RET*); MAP, *MUTYH*-associated polyposis (i.e., biallelic *MUTYH*); MEN1, multiple endocrine neoplasia type 1 (i.e., *MEN1*); NF2, neurofibromatosis type 2 (i.e., *NF2*); VHL, Von Hippel-Lindau syndrome (i.e., *VHL*). HCS, Hereditary Cancer Syndrome; GGT, Germline Genetic Testing; PVs, Pathogenic/Likely Pathogenic variants.

The diagnostic yield per cancer type (excluding incidental findings) was: 26% (30/115) for colorectal cancer, 23% (15/64) for ovarian cancer, 20% (98/491) for breast cancer and 11% (11/99) for other cancer in adults (Fig. 2). All corresponded to autosomal dominant syndromes, except for one *MUTYH*-associated polyposis (MAP) of autosomal recessive inheritance.

HBOC due to PVs in *BRCA1*/*2* genes accounted for 12% (60/491) of breast cancer and 17% (11/64) of ovarian cancer cases in our cohort (Fig. 2A, C), representing 61% (60/98) and 73% (11/15) of inherited cases, respectively. PVs in HR-DDR genes comprised 6% (28/491) of all breast cancer cases, explaining 29% (28/98) of the inherited ones (Fig. 2A). *ATM* was the only HR-DDR gene that contributed to other cancers (Fig. 1C). LS was the most prevalent among colorectal cancer cases, explaining 18% (21/115) of all cases in the cohort and 70% (21/30) of inherited ones (Fig. 2B), followed by FAP. The prevalence of rarer syndromes by cancer type is shown in Fig. 2.

### Performance of different GGT criteria for patients with breast cancer

Table 3 shows the frequency of *BRCA1/2* (jointly and separately) and non-*BRCA* PVs in breast cancer genes in 491 affected cases categorized by different GGT criteria. Most cases met the criteria of being diagnosed at ≤45 years (52.5%; 258/491), followed by cases ≥51 years (29.5%; 145/491) that met other inclusion criteria for HBOC (i.e., family history or TNBC subtype) or for other HCS. Near 24% (117/491) of the cases were referred due to TNBC subtype, 9% of which were diagnosed after 60 years and met other GGT criteria. Overall, 12% (59/491) were patients with bilateral disease, and less than 1% corresponded to affected males (0.8%; 4/491).

**Table 3:** Prevalence of PVs in breast cancer patients, according to different criteria defined in international guidelines.

HBOC criteria (*n* = 491)	Total per group		Non-TNBC		No family history of one or more affected relatives before 51 years		Non-TNBC & no family history of one or more affected relatives before 51 years		With family history of one or more affected relatives before 51 years		TNBC subtype	
Carriers	*BRCA1/2* (%; *n*)	Other Genes	*BRCA1/2* (%; *n*)	Other Genes	*BRCA1/2* (%; *n*)	Other Genes	*BRCA1/2* (%; *n*)	Other Genes	*BRCA1/2* (%; *n*)	Other Genes	*BRCA1/2* (%; n)	Other Genes
	*BRCA1* (%; *n*)	(%; *n*)	*BRCA1* (%; *n*)	(%; *n*)	*BRCA1* (%; *n*)	(%; *n*)	*BRCA1* (%; *n*)	(%; *n*)	*BRCA1* (%; *n*)	(%; n)	*BRCA1* (%; *n*)	(%; *n*)
	*BRCA2* (%; *n*)		*BRCA2* (%; *n*)		*BRCA2* (%; *n*)		*BRCA2* (%; *n*)		*BRCA2* (%; *n*)		*BRCA2* (%; *n*)	
Breast cancer ≤ 36 years (*n* = 81) (16.5%)	26% (21/81)		23% (14/61)		14.3% (3/21)		7.1% (1/14)		25% (10/40)		35% (7/20)	
	*BRCA1/2* (18.5%; 15/81)	Other Genes (7.4%; 6/81)	*BRCA1/2* (14.8%; 9/61)	Other Genes (8.2%; 5/61)	*BRCA1/2* (14.3%; 3/21)	Other Genes (0%; 0/21)	*BRCA1/2* (7.1%; 1/14)	Other Genes (0%; 0/14)	*BRCA1/2* (20%; 8/40)	Other Genes (5%; 2/40)	*BRCA1/2* (30%; 6/20)	Other Genes (5%; 1/20)
	*BRCA1* (12.3%; 10/81)		*BRCA1* (6.6%; 4/61)		*BRCA1* (9.5%; 2/21)		*BRCA1* (0%; 0/14)		*BRCA1* (12.5%; 5/40)		*BRCA1* (30%; 6/20)	
	*BRCA2* (6.2%; 5/81)		*BRCA2* (8.2%; 5/61)		*BRCA2* (4.8%; 1/21)		*BRCA2* (7.1%; 1/14)		*BRCA2* (7.5%; 3/40)		*BRCA2* (0%; 0/20)	
Breast cancer ≤ 45 years (*n* = 258) (52.5%)	19% (49/258)		15% (30/200)		19.7% (13/66)		13.7% (7/51)		18.9% (24/127)		32.8% (19/58)	
	*BRCA1/2* (12%; 31/258)	Other Genes (7%; 18/258)	*BRCA1/2* (8%; 16/200)	Other Genes (7%; 14/200)	*BRCA1/2* (15.2%; 10/66)	Other Genes (4.5%; 3/66)	*BRCA1/2* (9.8%; 5/51)	Other Genes (3.9%; 2/51)	*BRCA1/2* (11%; 14/127)	Other Genes (7.9%; 10/127)	*BRCA1/2* (26%; 15/58)	Other Genes (7%; 4/58)
	*BRCA1* (7.4%; 19/258)		*BRCA1* (2.5%; 5/200)		*BRCA1* (7.6%; 5/66)		*BRCA1* (0%; 0/51)		*BRCA1* (7.9%; 10/127)		*BRCA1* (24.1%; 14/58)	
	*BRCA2* (4.7%; 12/258)		*BRCA2* (5.5%; 11/200)		*BRCA2* (7.6%; 5/66)		*BRCA2* (9.8%; 5/51)		*BRCA2* (3.1%; 4/127)		*BRCA2* (2%; 1/58)	
Breast cancer between 46 to 50 years (*n* = 88) (17.9%)	16% (14/88)		15.2% (10/66)		22.2% (4/18)		28.6% (4/14)		15.6% (7/45)^a^		18.2% (4/22)	
	*BRCA1/2* (10.2%; 9/88)	Other Genes (5.7%; 5/88)	*BRCA1/2* (10.6%; 7/66)	Other Genes (4.5%; 3/66)	*BRCA1/2* (11.1%; 2/18)	Other Genes (11.1%; 2/18)	*BRCA1/2* (14.3%; 2/14)	Other Genes (14.3%; 2/14)	*BRCA1/2* (8.9%; 4/45)	Other Genes (6.7%; 3/45)	*BRCA1/2* (9.1%; 2/22)	Other Genes (9.1%; 2/22)
	*BRCA1* (2.3%; 2/88)		*BRCA1* (1.5%; 1/66)		*BRCA1* (0%; 0/18)		*BRCA1* (0%; 0/14)		*BRCA1* (2.2%; 1/45)		*BRCA1* (4.5%; 1/22)	
	*BRCA2* (8.0%; 7/88)		*BRCA2* (9.1%; 6/66)		*BRCA2* (11.1%; 2/18)		*BRCA2* (14.3%; 2/14)		*BRCA2* (6.7%; 3/45)		*BRCA2* (4.5%; 1/22)	
Breast cancer ≥ 51 years (*n* = 145) (29.5%)	24.1% (35/145)		19.4% (21/108)		8.8% (3/34)		8.3% (2/24)		32.1% (25/78)^b^		37.8% (14/37)	
	*BRCA1/2* (13.8%; 20/145)	Other Genes (10.3%; 15/145)	*BRCA1/2* (10.2%; 11/108)	Other Genes (9.3%; 10/108)	*BRCA1/2* (2.9%; 1/34)	Other Genes (5.9%; 2/34)	*BRCA1/2* (4.2%; 1/24)	Other Genes (4.2%; 1/24)	*BRCA1/2* (19.2%; 15/78)	Other Genes (12.8%; 10/78)	*BRCA1/2* (24.3%; 9/37)	Other Genes (13.5%; 5/37)
	*BRCA1* (3.4%; 5/145)		*BRCA1* (1.9%; 2/108)		*BRCA1* (0%; 0/34)		*BRCA1* (0%; 0/24)		*BRCA1* (5.1%; 4/78)		*BRCA1* (8.1%; 3/37)	
	*BRCA2* (10.3%; 15/145)		*BRCA2* (8.3%; 9/108)		*BRCA2* (2.9%; 1/34)		*BRCA2* (4.2%; 1/24)		*BRCA2* (14.1%; 11/78)		*BRCA2* (16.2%; 6/37)	
TNBC ≤ 60 years (*n* = 107) (21.8%)	31.8% (34/107)		NA		27% (7/26)		NA		38.8% (19/49)		NA	
	*BRCA1/2* (21.5%; 23/107)	Other Genes (10.3%; 11/107)	NA	NA	*BRCA1/2* (19.2%; 5/26)	Other Genes (7.7%; 2/26)	NA	NA	*BRCA1/2* (22.4%; 11/49)	Other Genes (16.3%; 8/49)	NA	NA
	*BRCA1* (15%; 16/107)				*BRCA1* (19.2%; 5/26)				*BRCA1* (12.2%; 6/49)			
	*BRCA2* (6.5%; 7/107)				*BRCA2* (0%; 0/26)				BRCA2 (10.2%; 5/49)			
TNBC ≥ 61 years (*n* = 10) (2.0%)	30% (3/10)^c^		NA		0% (0/3)		NA		42.9% (3/7)		NA	
	*BRCA1/2* (30%; 3/10)	Other Genes (0%; 0/10)	NA	NA	*BRCA1/2* (0%; 0/3)	Other Genes (0%; 0/3)	NA	NA	*BRCA1/2* (42.9%; 3/7)	Other Genes (0%; 0/7)	NA	NA
	*BRCA1* (20%; 2/10)				*BRCA1* (0%; 0/3)				*BRCA1* (28.6%; 2/7)			
	*BRCA2* (10%; 1/10)				*BRCA2* (0%; 0/3)				*BRCA2* (14.3%; 1/7)			
Bilateral breast cancer (*n* = 59) (12.0%)	30.5% (18/59)		21.4% (9/42)^d^		22.2% (4/18)		21.4% (3/14)		30.4% (7/23)		52.9% (9/17)^e^	
	*BRCA1/2* (16.9%; 10/59)	Other Genes (13.6%; 8/59)	*BRCA1/2* (9.5%; 4/42)	Other Genes (11.9%; 5/42)	*BRCA1/2* (16.7%;3/18)	Other Genes (5.6%; 1/18)	*BRCA1/2* (14.3%; 2/14)	Other Genes (7.4%; 1/14)	*BRCA1/2* (13%; 3/23)	Other Genes (17.4%; 4/23)	*BRCA1/2* (35.3%; 6/17)	Other Genes (17.6%; 3/17)
	*BRCA1* (8.5%; 5/59)		*BRCA1* (0%; 0/42)		*BRCA1* (5.6%; 1/18)		*BRCA1* (0%; 0/14)		*BRCA1* (8.7%; 2/23)		*BRCA1* (29.4%; 5/17)	
	*BRCA2* (8.5%; 5/59)		*BRCA2* (9.5%; 4/42)		*BRCA2* (11.1%; 2/18)		*BRCA2* (14.3%; 2/14)		*BRCA2* (4.3%; 1/23)		*BRCA2* (5.9%; 1/17)	
Male breast cancer (*n* = 4) (0.8%)	75% (3/4)^f^		NA		NA		NA		NA		NA	
	*BRCA1/2* (50%; 2/4)	Other Genes (25%; 1/4)	NA	NA	NA	NA	NA	NA	NA	NA	NA	NA
	*BRCA1* (0%; 0/4)											
	*BRCA2* (50%; 2/4)											

*PVs* Pathogenic/Likely Pathogenic variants, *TNBC* Triple-negative breast cancer.

The non-TNBC criteria refers to all subtypes other than triple-negative breast cancer (includes unspecified and missing data).

The family history criteria (one or more before age 51) refer to family history of cancer in general, with at least one diagnosed at 50 years-old or before

^a^27/45 cases with family history (one or more before age 51) correspond to cases with a family history of breast/ovarian cancer. Of these, 22% (6/27) are carriers of PVs in risk genes.

^b^49/78 cases with family history (one or more before age 51) correspond to cases with a family history of breast/ovarian cancer. Of these, 34.7% (17/49) are carriers of PVs in risk genes.

^c^Among the 10 TNBC cases over 60 years old, nine reported family history (one or more before age 51 (*n* = 7) or at any age (*n* = 2)) and one without family history had bilateral presentation. In this group, overall, 4/10 had bilateral presentation with or without family history.

^d^Excludes TNBC in the first or second breast cancer. If only TNBC in the first breast cancer is excluded, the prevalence of germline PVs would be 25% (12/48).

^e^TNBC in the bilateral group includes TNBC in the first or second breast cancer.

^f^Male breast cancer cases with PVs corresponded to two *BRCA2* and one *RAD51D* carriers.

Among breast cancer cases, also affected with ovarian cancer, 40% (4/10) corresponded to carriers of PV in the following genes (one of each): *BRCA1*, *BRCA2*, *ATM* and *CHEK2*.

**Fig. 3:**
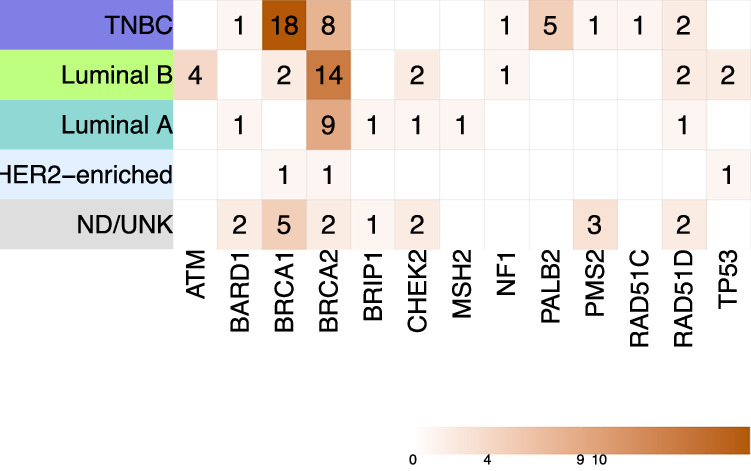
Distribution of pathogenic/likely pathogenic variants (PVs) in breast cancer genes among the different molecular subtypes of breast cancer cases (*n* = 98). Heatmap displaying the distribution of PVs identified in genes with known evidence of association with breast cancer risk among different molecular subtypes, using the St. Gallen 2013 surrogate classification. Incidental findings are not included in this heatmap. TNBC, triple negative breast cancer subtype; ND/UNK, cases with missing data regarding one or more markers; PVs, pathogenic/likely pathogenic variants.

The multinomial logistic regression model (Table 4) supports that TNBC subtype [OR 2.99; 95%CI (1.79–4.98); *p*-value < 0.001] and bilateral presentation [OR 2.24; 95%CI (1.16–4.34); *p*-value 0.02] are the best predictors of an inherited condition, regardless of the age at diagnosis or family history (as measured here). Similar results were found for predicting carrier status of PVs in *BRCA1/2* genes [OR 3.54, *p*-value < 0.001; and OR 2.04, *p*-value 0.08, respectively] and non-*BRCA* genes [OR 2.18, *p*-value 0.05; and OR 2.51, *p*-value 0.05]. *BRCA1* carrier status was mainly associated with TNBC subtype [OR 16.24, *p*-value < 0.001] and early age at diagnosis [OR 0.95; *p*-value 0.05]. The distribution of PVs among the different breast cancer molecular subtypes is shown in Fig. 3.

**Table 4:** Multinomial logistic regressions for prediction of breast cancer syndromes and carrier status (*BRCA* and non-*BRCA*).

Patient characteristics	OR	*p*-value (95% CI)
Is a hereditary breast cancer syndrome [Ref = No]		
Age at diagnosis	1	0.78 (0.98–1.02)
*Bilateral presentation*	**2.24**	**0.02 (1.16**–**4.34)**
TNBC subtype	**2.99**	**<0.001 (1.79**–**4.98)**
Positive family history (one or more <51years)	1.46	0.19 (0.82 - 2.57)
*Carrier status [Ref = Not a carrier]*		
Age at diagnosis		
*BRCA1/2* genes	0.99	0.98 (0.97–1.03)
Non-*BRCA* genes	1	0.72 (0.97–1.04)
Bilateral presentation		
*BRCA1/2* genes	2.04	0.08 (0.91– 4.58)
*Non-BRCA genes*	**2.51**	**0.05 (0.99**–**6.30)**
TNBC subtype		
*BRCA1/2* genes	**3.54**	**<0.001 (1.94**–**6.47)**
Non-*BRCA* genes	**2.18**	**0.05 (0.99**–**4.81)**
Positive family history (one or more <51years)		
*BRCA1/2* genes	1.34	0.40 (0.67–2.67)
Non-*BRCA* genes	1.6	0.28 (0.67–3.78)
*BRCA1 carrier status [Ref* *=* *No PVs detected]*		
Age at diagnosis	**0.95**	**0.05 (0.91**–**0.99)**
Bilateral presentation	2.27	0.16 (0.71–7.20)
TNBC subtype	**16.24**	**<0.001 (5.28**– **49.89)**
Positive family history (one or more <51years)	1.49	0.44 (0.53–4.21)
*BRCA2 carrier status [Ref* *=* *No PVs detected]*		
Age at diagnosis	1.03	0.08 (0.99–1.07)
Bilateral presentation	1.27	0.65 (0.45–3.60)
TNBC subtype	0.97	0.94 (0.42–2.26)
Positive family history (one or more <51years)	1.11	0.82 (0.46–2.65)

*PVs* Pathogenic/Likely Pathogenic variants, *TNBC* Triple-negative breast cancer.

Bold values indicate *p*-values ≤ 0.05.

### Recurrent germline PVs in high-risk Colombian patients with cancer

Only 3/14 different PVs in *BRCA1* gene were recurrent, while for *BRCA2* recurrent PVs were 9/18 (Table 1 and Supplemental Figure S6). No CNVs were found in *BRCA1* in our cohort. Conversely, two CNVs were identified in *BRCA2*, including a recurrent large deletion of exons 1 to 14 detected in five unrelated patients with breast or ovarian cancer. An *ATM* gene deletion of exons 27 to 28 was detected in four unrelated patients affected with colorectal, ovarian, and prostate cancers. The nonsense PV *BARD1:c.2229dup p.(Asn744Ter)* was detected in four unrelated patients with breast cancer. Two different PVs in *PALB2* were detected exclusively in patients with TNBC, with *PALB2:c.2288_2291del p.(His762_Leu763insTer)* being found in four of these. Other unrelated patients with breast cancer were carriers of *RAD51D:c.556* *C* > *T p.(Arg186Ter)* (*n* = 3) or *RAD51D:c.94_95del p.(Val32PhefsTer38)* (*n* = 5). *MLH1* gene PVs were identified only in colorectal cancer cases, and the most frequent were *MLH1:c.1918C>T p.(Pro640Ser)* (*n* = 4) and *MLH1:c.790+1* *G* > *A p.?* (*n* = 5). The PV *PMS2:c.2182_2184delinsG p.(Thr728AlafsTer7)* was detected in six unrelated patients with atypical clinical phenotypes, and these were confirmed at an external laboratory given the presence of pseudogenes. The complete list of recurrent PVs in our cohort is specified in bold in Table 1.

## Discussion

Colombian regions exhibit genetic admixture, with contributions from European, African, and Indigenous American ancestral populations [9–11]. Our study on actionable germline findings in patients with cancer addresses a gap in genetic research, which frequently centers on populations of European ancestry [8], underscoring the necessity to understand genetic diversity in tackling health disparities [11, 23].

With the program, pre- and post-test GC appointments increased by 2.6 times compared to the previous year (2017 = 263 vs 2018 = 638; 2019 = 780; I-2020 = 598) (data not shown). The majority were young female patients with breast cancer, underlining its significance in Colombian women [24] and supporting referral criteria based on age at diagnosis. Most patients met International GGT criteria and underwent testing (Supplemental Fig. S3 and Tables S4-S5). Despite the rise in consultations, our study underscores persistent disparities in service access, with most participants coming from large urban areas. Educational initiatives are needed to expand GC to remote and underserved areas in Colombia [2].

PVs prevalence among patients with cancer varies widely (3% to 30.7%) due to factors like population studied, panel size, selection criteria, and cancer type [20, 25–27]. Using multigene panels increases incidental findings and identification of carriers of variants associated with recessive syndromes. We report a 26% prevalence of PVs and a 21% prevalence of HCS in a high-risk cohort of 769 Colombian adults with solid malignancies tested with a 105-gene panel. Similar studies on programs offering GC and GGT based on personal or family history of cancer, or clinical judgment, reported HCS prevalence of 18.1%, 21.8%, and 39.7% in Brazil, Eastern Spain, and Mexico, respectively, using NGS panels [28–30].

Population-specific differences in the prevalence of *BRCA1/2* PVs have been reported in Latin American countries (3.0 to 47.8%), with lower prevalence among unselected breast cancer cases and higher prevalence among individuals at-high-risk of HBOC syndrome [4]. Few studies on Colombian breast/ovarian cancer cases have reported a yield of PVs of 11% to 22% on selected cases based on international criteria and using multigene cancer panels (25 to 143 genes), with *BRCA1/2* genes contributions between 7.2% to 17.6% [31–33]. In our study, 20% (113/555) of the breast and ovarian cancer cases were identified as carriers of PVs in associated genes, with major contributions from *BRCA1* and *BRCA2* genes (12% and 17%, respectively). The higher proportion of *BRCA2* PVs in some populations has been previously reported in other studies in selected or unselected women with breast cancer from Colombia [31, 34] and US Latinos from Puerto Rico and Cuba [7], whereas *BRCA1* PVs are more frequent in breast and ovarian patients from Peru [3] and US Latinos from Mexico [7].

Our study identifies relevant contributions of *NF1* and *TP53* genes to inherited breast cancer in Colombian women, in addition to HR-DDR genes reported by us and others [31–34]. In our cohort, luminal B (35.2%) and TNBC (24.4%) were the most frequent subtypes; although, TNBC exhibited the highest diagnostic yield of a HCS with (31.6%). Luminal B’s prevalence aligns with prior reports in Colombian women [35]. Consistent with other studies [36], we report genotype-phenotype correlations between TNBC subtype and PVs in *BRCA1* and a tendency with non-*BRCA* genes (i.e., *PALB2*, *RAD51D*). *ATM* and *CHEK2* PVs carriers were exclusively estrogen receptor-positive, while *TP53* PVs were only observed in non-TNBC.

LS prevalence among Hispanic/Latino populations in the U.S. is around 17.9%, with the highest rates in immigrants from Mexico, El Salvador, and Guatemala ( ~ 30%), compared to those from other Latin American countries ( ~ 8.0%) [37]. Our study contributes to characterizing LS in Latin America [38–42]. We found that most LS cases were associated with *MLH1* (46.7%) and *PMS2* (30.0%), while *MSH6* (13.3%) and *MSH2* (10.0%) were less common. Other LS studies in Latin America have shown similar contributions from *MLH1* (43% – 54%) and *MSH6* (4% – 10%), but higher *MSH2* (32% – 43%) and lower *PMS2* (0.8% – 10%) [40–42]. Our study did not identify *EPCAM* large deletions among LS cases, although its contribution seems variable [38, 39, 42]. This variable contribution of PVs in MMR genes across Latin America highlights the genetic diversity within our admixed populations, underscoring the need for tailored GC and GGT approaches. HCS known to be less prevalent, were detected in a few cancer cases in our high-risk cohort, highlighting the need for multicenter cohorts to enhance detection.

Our study reports 33 recurrent PVs among this high-risk Colombian cohort, alongside a previously identified recurrent *SDHB* alteration (ex1del) that explains 42% of inherited PGL/PCC cases, prompting the recommendation for routine MLPA evaluation of SDHx genes in these patients in Colombia [20]. The majority of recurrent alterations found in three or more carriers are well-known PVs in *BRCA1 [c.1674del p.(Gly559ValfsTer13), c.3331_3334del p.(Gln1111AsnfsTer5), and c.5123* *C* > *A p.(Ala1708Glu)]* [4, 33, 43, 44] and *BRCA2 [c.1763_1766del p.(Asn588SerfsTer25), c.6024dup p.(Gln2009AlafsTer9), and c.4889* *C* > *G p.(Ser1630Ter)]* [6, 33, 44]. The *BRCA2* ex1-14del was reported in two Colombian families that shared a conserved haplotype implying that this CNV may have arisen from a common founder [6], and in this study we detected the same CNV in five additional independent cases. Other recurrent PVs detected in our cohort have been reported in a collaborative publication with Invitae [33], since at the beginning of the program we used an external laboratory to test 54 patients with breast cancer and 10 with colorectal cancer, and positive cases were later confirmed with our sequencing protocol. For example, *PALB2:c.2288_2291del p.(His762_Leu763insTer)* was previously found in five Colombians (including one from our cohort) [33], and we are reporting the same PV in three additional unrelated cases, all TNBC. Two PVs in *RAD51D [c.556* *C* > *T p.(Arg186Ter) and c.94_95del p.(Val32PhefsTer38)]*, were reported previously in one Colombian patient each [33], and we found these to be recurrent in our Colombian cohort. The *ATM* ex27-29del reported before [33], was not detected in our study, although we found a similar partial deletion in *ATM* comprising exons 27-28 in four unrelated patients affected with colorectal, ovarian, and prostate cancer.

Other approaches, such as universal GGT [45, 46] or tumor genetic profiling [47] in routine oncology practice, have been shown to increase the detection of PVs associated with HCS, compared to the restricted GGT criteria in current guidelines. However, in Colombia, limited resources hinder their widespread adoption. Demonstrating the long-term impact of identifying inherited cancer cases on clinical outcomes is crucial for justifying the allocation of resources and integrating these strategies into routine clinical practice. Two recent Colombian studies support these efforts: one analyzed 10 breast cancer genes in 400 unselected women with breast cancer, revealing a 6% prevalence of PVs and advocating for universal GGT [34]; the second, published by our group, assessed disease-free and overall survival rates in carriers identified through our Hereditary Cancer Program, categorizing them by PVs in breast cancer-associated or non-associated genes [48]. These studies provide key insights that could prompt further research and reevaluation of these approaches in Colombia’s unique healthcare context.

This study has limitations. Implementing a unified database in our program presented challenges in capturing specific clinical and pathological variables for each cancer type. The use of summarized variables to record family history may have further restricted the depth of analysis. Additionally, the predominantly sampled population from the Andean region, adjacent Plains, and Caribbean Coasts limited the generalizability of findings due to insufficient representation from remote areas, such as the southeastern Plains and Pacific Coasts. Cascade testing was feasible for only a fraction of participant’s relatives, with broader implementation constrained by financial limitations, the availability of assays for specific PVs, inconsistent insurance coverage, and patients’ reluctance to share genetic information with family members.

Current efforts by the INC-C to improve genetic characterization for HCS nationwide include enhancing access to GC through telemedicine and social worker support. Additionally, the INC-C is advocating for the creation of a National Hereditary Cancer Network to ensure broader and more equitable access to genetic services. These initiatives aim to unify regional efforts, strengthen research collaborations, and reduce disparities across the country. To address barriers to cascade testing, we are standardizing cost-effective testing assays in our laboratories by adopting technologies complementary to NGS. We have also introduced a system of letters directed to relatives and insurance providers to encourage participation in GC and GGT, supported by the social work team, which assists in contacting, recontacting, and ensuring continued patient engagement.

In conclusion, this report presents the first comprehensive analysis of HCS in a high-risk cohort of Colombian patients with cancer, highlighting actionable and recurrent PVs. By leading the largest Hereditary Cancer Program in the country, we have advanced our understanding of germline PVs in oncology and established an Institutional Registry that captures socio-demographic, clinic-pathologic, and genetic data for selected patients referred to the program. This resource makes a significant contribution to characterizing the molecular epidemiology of HCS in Colombians, beyond HBOC, and may offer insights relevant to other Latin American populations with similar risk profiles.

## Supplementary information

Supplemental Material

## Data Availability

The datasets generated and analyzed during this study are not publicly available because all cases were enrolled in the program under informed consent provided to the National Tumor Biobank Terry Fox (BNTTF, in Spanish: Banco Nacional de Tumores Terry Fox) of the Instituto Nacional de Cancerología (INC-C) in Colombia, which serves as the custodian of the data. In line with recent Colombian regulations (Law 2287 of 2023), biobanks may grant access to de-identified data, including raw data such as FASTQ and VCF files, for research purposes. Access will be granted upon a reasonable request, subject to review and approval by the ethics committee, and strict compliance with the participants’ signed informed consent. Requests should be directed to the BNTTF [email: bnttf@cancer.gov.co].
