# Supplementary material for: Building a hereditary cancer program in Colombia: analysis of germline pathogenic and likely pathogenic variants spectrum in a high-risk cohort
Source: Eur J Hum Genet. 2025 Mar 10;33(7):913–29. doi: 10.1038/s41431-025-01807-y (PMC12229586; doi:10.1038/s41431-025-01807-y)
Supplement: Supplementary file 1 — Supplemental Material [file 41431_2025_1807_MOESM1_ESM.pdf]

Building a Hereditary Cancer Program in Colombia: Analysis of Germline Pathogenic and Likely Pathogenic Variants Spectrum in a High-Risk Cohort

María Carolina Sanabria-Salas PhD, Ana Lucía Rivera-Herrera MSc, María Carolina Manotas MD, Gonzalo Guevara MD, Ana Milena Gómez MD, Vilma Medina MSc, Sandra Tapiero MSc, Antonio Huertas MSc, Marcela Nuñez MSc, Miguel Zamir Torres MSc, Julián Riaño-Moreno PhD, Rafael Parra-Medina PhD, Juan Carlos Mejía MD, Luis G. Carvajal-Carmona PhD.

Supplementary Information

1. Supplementary Methods:

1.1. Conception of the Hereditary Cancer Program at the INC-C
1.2. Developer team and construction of the Program´s Registry
1.3. High throughput sequencing, bioinformatics pipeline and genetic variant interpretation
1.4. Multiplex Ligation-Dependent Probe Amplification (MLPA) of *BRCA1* and *BRCA2* genes

2. Supplementary Tables:

Table S1. Defined purposes for the expansion of the Hereditary Cancer Program at the INC-C
Table S2. Genes included in the multigene panel
Table S3. Place of birth and domicile of all the patients registered in the Hereditary Cancer Program at the INC-C (n = 986)
Table S4. Clinical and demographic characteristics of patients diagnosed with breast, colorectal, and ovarian cancer
Table S5. Characteristics of patients diagnosed with other cancers

3. Supplementary Figures:

Figure S1. Place of birth reported by all the patients registered in the Hereditary Cancer Program at the INC-C (n = 986)
Figure S2. Domicile reported by all the patients registered in the Hereditary Cancer Program at the INC-C (n = 986)
Figure S3. Age at diagnosis and sex distribution by cancer type of all the patients registered in the Hereditary Cancer Program at the INC-C (n = 986)
Figure S4. Mismatch repair (MMR) immunohistochemistry (IHC) patterns and corresponding pathogenic/likely pathogenic variants (PVs) in patients with colorectal cancer (n = 115)
Figure S5. Prevalence of hereditary cancer syndromes (HCS) calculated for adult cancer cases at risk and with genetic test results (n = 769)
Figure S6. Distribution and effect of pathogenic/likely pathogenic variants (PVs) in *BRCA1* and *BRCA2* genes

1. Methods:

1.1. Conception of the Hereditary Cancer Program at the INC-C

The Scientific Committee of the Instituto Nacional de Cancerología, Colombia (INC-C), approved the implementation of a Hereditary Cancer Program in 2017 as a Quality Improvement Project to offer germline genetic testing as a standard of care for patients with cancer who may have an underlying inherited condition. The institutional departments or groups (and professionals) involved in the conception and expansion of the program were the National Tumor Biobank Terry Fox (BNTTF, in Spanish: Banco Nacional de Tumores Terry Fox) (A.H., J.R.-M., R.P.-M., J.C.M.), the Molecular Pathology in Oncology & Genetics Department (GGP) and the Cancer Biology Research Group (M.C.S.-S.). The proposal focused on acting on three main axes or purposes described in Supplemental Table S1.

1.2. Developer team and construction of the Program´s Registry

Three geneticists (M.C.S.-S., G.G., A.M.G.) performed pre and post-test genetic counseling, and four biologists with training in genetics (A.L.R.-H., V.M., S.T., A.H.) supported different activities of the program, such as the registration of the patients and their related information in the database designed for the program and the performance of genetic testing assays. A fourth medical geneticist (M.C.M.) performed an automated and manual curation of all the germline genetic variants included in the registry [1]. The revised program´s registry or database includes sociodemographic, clinical, pathological, and germline genetic information of the cohort of patients referred for genetic counseling through the Hereditary Cancer Program at the INC-C, between April 2018 and June 2020. The information was entered by A.L.R.-H. and reviewed by M.C.S.-S. before the analysis, using the curated germline genetic variant report [1]. Study variables are sex, age at the first genetic consultation, type of cancer or tumor, age at diagnosis, second primary cancer, place of birth, place of residence, molecular somatic results (i.e. breast cancer intrinsic subtype recorded based on St. Gallen 2013 surrogates, mismatch repair proteins immunohistochemistry and *BRAF* mutation status, if applicable), family history of related cancers or tumors in first-, second- and third-degree relatives, if a germline genetic testing was performed, type of genetic testing, results of the germline genetic testing, classification of the genetic variant and its reclassification, and if a hereditary cancer syndrome was diagnosed, specifying the name of the syndrome and the inheritance mode.

1.3. High throughput sequencing, bioinformatics pipeline and genetic variant interpretation

Details on germline DNA extraction, library preparation, sequencing assays, variant calling, and genetic data interpretation are available in previous publications [1, 2]. Briefly, the FastQC files were aligned to the hg19 human reference genome with the Burrows–Wheeler Aligner (BWA) tool. Variant calling and annotation of single nucleotide variants (SNVs) and short insertions and deletions (INDELs) were carried out with the SOPHiA DDM® platform using the ILL1IC1G3_TSC algorithm (Sophia Genetics, Saint-Sulpice, Switzerland). This algorithm also allows inferring copy number variations (CNV) from sequence data for all genes included in the 105-cancer gene panel, and the detection of ALU elements. The Human Genome Variation Society (HGVS) nomenclature (http://www.hgvs.org/) was used in the genetic report, and the five-tier criteria of the American College of Medical Genetics and Genomics (ACMG) for variant classification as pathogenic (P), likely pathogenic (LP) [jointly referred as PVs], variant of uncertain significance (VUS), likely benign (LB), and benign (B) were implemented [3]. We conducted an automated and manual curation of all the germline genetic variants included in the registry and used these updated interpretations in our analysis [1]. SNVs and INDELs categorized as PVs and reported in this study were verified with the variant description validation software, VariantValidator [4]. Finally, when needed for confirmation purposes, Sanger sequencing and Multiplex Ligation-Dependent Probe Amplification (MLPA) were used as orthogonal methods, to detect specific SNV and short INDELs, or large deletions or duplications, respectively. These assays were conducted at external laboratories for most genes, except for *BRCA1* and *BRCA2* MLPAs which were standardized at our laboratory.

1.4. Multiplex Ligation-Dependent Probe Amplification (MLPA) of *BRCA1* and *BRCA2* genes

Briefly, the probe mixes MLPA P002 and P087 were used to screen *BRCA1* and the probe mixes MLPA P090 and P077 to screen *BRCA2*, according to the instructions provided by the manufacturer (MRC-Holland, Amsterdam, The Netherlands). Multiplex PCR-amplified products were separated by capillary gel electrophoresis in a 3500 Genetic Analyzer 8-Capillary Array (Applied Biosystems™, United States). The software Coffalyser v.210604.1451 (MRC-Holland, Amsterdam, The Netherlands) was used to analyze copy number variants.

2. Supplementary Tables:

Table S1. Defined purposes for the expansion of the Hereditary Cancer Program at the INC-C

Purpose	Overall Result
i) Strengthening the Genetic and Molecular Pathology in Oncology laboratories in state-of-the-art molecular techniques for the study of germline variants (NGS and MLPA).	Generation of local genetic data to increase the knowledge of common and rare genetic variants in Colombians, reducing bias related to missing diversity in human genetic studies and avoiding unfavorable implications for risk prediction of diseases in our underrepresented Latin American population [5].
ii) Training oncologists, oncology surgeons, and other professionals involved in the program on key genetic counseling concepts and international criteria for identifying patients with cancer who are at high-risk and require genetic counseling.	Both referrals to genetic counseling and detection of PVs increased, meaning more patients with cancer are receiving specific screening and cancer prevention recommendations, as part of their comprehensive management and in coordination with other health care services.
iii) Implementing a database to register all the cases referred to genetic counseling at the INC-C.	Creation of a Registry in a Research Electronic Data Capture (REDCap) platform with socio-demographic, clinic-pathologic and genetic information from every patient seen by the genetic service. This Institutional Registry not only is supporting clinical follow-up of the patients, but also represents an invaluable tool for generating epidemiological reports relevant for the Latin American community.

Table S2. Genes included in the multigene panel

AIP	*ALK*	*APC*	*ATM*	*BAP1*	*BARD1*	*BLM*	*BMPR1A*
BRCA1	*BRCA2*	*BRIP1*	*BUB1B*	*CASR*	*CDC73*	*CDH1*	*CDK4*
CDKN1B	*CDKN1C*	*CDKN2A*	*CEBPA*	*CEP57*	*CHEK2*	*CYLD*	*DDB2*
DICER1	*DIS3L2*	*EGFR*	*EPCAM*	*ERCC2*	*ERCC3*	*ERCC4*	*ERCC5*
EXT1	*EXT2*	*EZH2*	*FANCA*	*FANCB*	*FANCC*	*FANCD2*	*FANCE*
FANCF	*FANCG*	*FANCI*	*FANCL*	*FANCM*	*FH*	*FLCN*	*GATA2*
GNAS	*GPC3*	*HNF1A*	*HRAS*	*KIT*	*MAX*	*MEN1*	*MET*
MLH1	*MRE11A*	*MSH2*	*MSH6*	*MUTYH*	*NBN*	*NF1*	*NF2*
NSD1	*PALB2*	*PDE4D*	*PHOX2B*	*PMS1*	*PMS2*	*POLD1*	*POLE*
PPM1D	*PRF1*	*PRKAR1A*	*PTCH1*	*PTEN*	*RAD50*	*RAD51C*	*RAD51D*
RB1	*RECQL4*	*RET*	*RHBDF2*	*RUNX1*	*SBDS*	*SDHA*	*SDHAF2*
SDHB	*SDHC*	*SDHD*	*SLX4*	*SMAD4*	*SMARCB1*	*STK11*	*SUFU*
TMEM127	*TP53*	*TSC1*	*TSC2*	*VHL*	*WRN*	*WT1*	*XPA*
XPC							

Table S3. Place of birth and domicile of all the patients registered in the Hereditary Cancer Program at the INC-C (n = 986)

Department, n (%)	Place of	
	Birth	Domicile
Amazonas	5 (0.52)	5 (0.51)
Antioquia	13 (1.36)	0 (0.00)
Arauca	6 (0.63)	10 (1.01)
Archipiélago De San Andrés, Providencia	2 (0.21)	2 (0.20)
Atlántico	8 (0.83)	4 (0.41)
**Bogotá DC**	**310 (32.3)**	**566 (57.4)**
Bolívar	12 (1.25)	5 (0.51)
**Boyacá**	**133 (13.8)**	**64 (6.49)**
Caldas	14 (1.46)	4 (0.41)
Caquetá	11 (1.15)	8 (0.81)
**Casanare**	**24 (2.50)**	**39 (3.96)**
Cauca	3 (0.31)	2 (0.20)
Cesar	12 (1.25)	5 (0.51)
Chocó	2 (0.21)	1 (0.10)
Córdoba	5 (0.52)	0 (0.00)
**Cundinamarca**	**112 (11.6)**	**81 (8.22)**
Guainía	0 (0.00)	0 (0.00)
Guaviare	1 (0.10)	1 (0.10)
Huila	31 (3.23)	12 (1.22)
La Guajira	12 (1.25)	9 (0.91)
Magdalena	18 (1.88)	15 (1.52)
**Meta**	**39 (4.07)**	**50 (5.07)**
Nariño	9 (0.94)	5 (0.51)
Norte De Santander	12 (1.25)	6 (0.61)
Putumayo	6 (0.63)	7 (0.71)
Quindío	3 (0.31)	0 (0.00)
Risaralda	6 (0.63)	1 (0.10)
Santander	36 (3.75)	6 (0.61)
Sucre	6 (0.63)	2 (0.20)
**Tolima**	**94 (9.80)**	**74 (7.51)**
Valle Del Cauca	13 (1.36)	0 (0.00)
Vaupés	1 (0.10)	1 (0.10)
Vichada	0 (0.00)	1 (0.10)
Total	959	986

Total patients registered in the REDCap database created for the Institutional Hereditary Cancer Program, from April 2018 to June 2020, amount to 986 unrelated cases. This table displays the frequencies of birthplace and domicile for all these cases across Colombian departments. Place of birth was recorded for 959 patients, other 27 had missing entries. These missing data likely correspond to immigrants from Venezuela receiving cancer treatment at the INC-C. Domicile was registered for all cases, indicating that most patients settle in Bogotá for treatment.

Table S4. Clinical and demographic characteristics of patients diagnosed with breast, colorectal, and ovarian cancer

Characteristic		All (n = 491)	Carriers of PVs[a] (n = 126)	Non-carriers[b] (n = 164)	p-value[c]	VUS (n = 201)
**Breast Cancer**						
**Age at diagnosis (years)**	Median [IQR]	45.0 [14.0]	45.5 [16.0]	46.0 [15.0]	0.575	44.0 [10.0]
**Age at diagnosis (years), n (%)**	≤ 50	346 (70.5)	81 (64.3)	110 (67.1)	0.621	155 (77.1)
	> 50	145 (29.5)	45 (35.7)	54 (32.9)		46 (22.9)
**Sex, n (%)**	Female	487 (99.2)	123 (97.6)	163 (100)	0.081	200 (99.5)
	Male	4 (0.80)	3 (2.40)	0 (0.00)		1 (0.50)
**Degree of invasion, n (%)**	In situ	11 (2.20)	2 (1.60)	5 (3.00)	0.795	4 (2.00)
	Infiltrating	470 (95.7)	118 (93.7)	158 (96.3)		194 (96.5)
	Missing data	10 (2.00)	6 (4.80)	1 (0.60)		3 (1.50)
**Histopathology[d], n (%)**	Ductal	422 (87.9)	112 (90.3)	138 (86.8)	**0.050**	172 (87.3)
	Lobular	16 (3.30)	1 (0.80)	8 (5.00)		7 (3.60)
	Mucinous	11 (2.30)	0 (0.00)	5 (3.10)		6 (3.00)
	Medullary	3 (0.60)	0 (0.00)	1 (0.60)		2 (1.00)
	Other	11 (2.30)	4 (3.20)	4 (2.50)		3 (1.50)
	Missing data	17 (3.50)	7 (5.60)	3 (1.90)		7 (3.60)
**Degree of differentiation[d], n (%)**	Well	34 (7.10)	4 (3.20)	16 (10.1)	**< 0.01**	14 (7.10)
	Moderate	240 (50.0)	47 (37.9)	87 (54.7)		106 (53.8)
	Poor	162 (33.8)	57 (46.0)	47 (29.6)		58 (29.4)
	Missing data	44 (9.20)	16 (12.9)	9 (5.70)		19 (9.60)
**Locations[d], n (%)**	Unilateral	426 (88.7)	104 (83.9)	142 (89.3)	0.281	180 (91.4)
	Bilateral	51 (10.6)	19 (15.3)	17 (10.7)		15 (7.60)
	Missing data	3 (0.60)	1 (0.80)	0 (0.00)		2 (1.00)
**Stage[d], n (%)**	Early	204 (42.5)	48 (38.7)	69 (43.4)	0.637	87 (44.2)
	Locally advanced	201 (41.9)	57 (46.0)	68 (42.8)		76 (38.6)
	Metastatic	44 (9.20)	9 (7.30)	16 (10.1)		19 (9.60)
	Missing data	31 (6.50)	10 (8.10)	6 (3.80)		15 (7.60)
**Molecular subtype[d], n (%)**	Luminal A	98 (20.4)	20 (16.1)	39 (24.5)	0.126	39 (19.8)
	Luminal B	169 (35.2)	38 (30.6)	53 (33.3)		78 (39.6)
	HER2-enriched	29 (6.04)	4 (3.23)	11 (6.92)		14 (7.11)
	Triple negative	117 (24.4)	41 (33.1)	39 (24.5)		37 (18.8)
	Inconclusive	42 (8.75)	9 (7.26)	13 (8.18)		20 (10.1)
	Missing data	25 (5.21)	12 (9.68)	4 (2.52)		9 (4.57)

IQR: Interquartile range; PVs: Pathogenic/Likely Pathogenic variants; VUS: variant of uncertain significance

a: Patients with any PV in any gene are included in this table

b: Non-carrier group is composed of individuals without any detectable PV or VUS (negative result)

c: Carriers of PVs vs non-carriers. Wilcox test, two-sided, was used for continuous variables (age). Fisher test was used for categorical variables.

d: Excluding 11 in situ cases (n = 480)

Molecular subtype surrogate classification was performed according to the St. Gallen 2013.

Table S4. Clinical and demographic characteristics of patients diagnosed with breast, colorectal, and ovarian cancer (continued)

Characteristic		All (n = 115)	Carriers of PVs[a] (n = 38)	Non-carriers[b] (n = 22)	p-value[c]	VUS (n = 55)
**Colorectal cancer**						
**Age at diagnosis (years)**	Median [IQR]	50.5 [24.5]	54.5 [18.0]	49.0 [33.8]	0.778	49.0 [25.5]
**Age at diagnosis (years), n (%)**	≤ 50	58 (50.4)	15 (39.5)	12 (54.5)	0.258	31 (56.4)
	> 50	57 (49.6)	23 (60.5)	10 (45.5)		24 (43.6)
**Sex, n (%)**	Female	63 (54.8)	19 (50.0)	13 (59.1)	0.496	31 (56.4)
	Male	52 (45.2)	19 (50.0)	9 (40.9)		24 (43.6)
**Histopathology, n (%)**	Adenocarcinoma	101 (87.8)	34 (89.5)	18 (81.8)	0.513	49 (89.1)
	Mucinous	10 (8.70)	3 (7.90)	4 (18.2)		3 (5.50)
	Signet ring cell	3 (2.60)	1 (2.60)	0 (0.00)		2 (3.60)
	Missing data	1 (0.90)	0 (0.00)	0 (0.00)		1 (1.80)
**Degree of differentiation, n (%)**	Well	17 (14.8)	4 (10.5)	5 (22.7)	0.409	8 (14.5)
	Moderate	76 (66.1)	27 (71.1)	12 (54.1)		37 (67.3)
	Poor	8 (7.00)	3 (7.90)	1 (4.80)		4 (7.30)
	Missing data	14 (12.2)	4 (10.5)	4 (19.0)		6 (10.9)
**Locations, n (%)**	Right	43 (37.4)	18 (47.4)	8 (36.4)	0.437	17 (30.9)
	Left	70 (60.9)	19 (50.0)	13 (59.1)		38 (69.1)
	Missing data	2 (1.70)	1 (2.60)	1 (4.50)		0 (0.00)
**Stage, n (%)**	Early	41 (37.5)	15 (39.5)	7 (31.8)	0.801	19 (34.5)
	Locally advanced	44 (38.3)	12 (31.6)	8 (36.4)		24 (43.6)
	Metastatic	20 (17.4)	5 (13.2)	4 (18.2)		11 (20.0)
	Missing data	10 (8.70)	6 (15.8)	3 (13.6)		1 (1.80)
**IHC for MMR proteins, n (%)**	All Intact	54 (46.9)	9 (23.7)	8 (36.4)	0.070	37 (67.2)
	MLH1/PMS2 Absent	16 (13.9)	10 (26.3)	1 (4.55)		5 (9.09)
	MLH1/PMS2/MSH6 Absent	2 (1.74)	2 (5.26)	0 (0.00)		0 (0.00)
	MSH2/MSH6 Absent	2 (1.74)	0 (0.00)	2 (9.09)		0 (0.00)
	MSH2/PMS2/MSH6 Absent	1 (0.87)	1 (2.63)	0 (0.00)		0 (0.00)
	MSH6 Absent	2 (1.74)	2 (5.26)	0 (0.00)		0 (0.00)
	PMS2 Absent	5 (4.35)	4 (10.5)	1 (4.55)		0 (0.00)
	Missing data	33 (28.7)	10 (26.3)	10 (42.8)		13 (23.6)
**IHC for MMR proteins, n (%)**	All Intact	54 (46.9)	9 (23.7)	8 (36.3)	0.858	37 (67.2)
	One or more absent	28 (24.3)	19 (50.0)	4 (18.2)		5 (9.09)
	Missing data	33 (28.7)	10 (26.3)	10 (45.5)		13 (23.6)

IQR: Interquartile range; PVs: Pathogenic/Likely Pathogenic variants; VUS: variant of uncertain significance; IHC: immunohistochemistry; MMR: mismatch repair
a: Patients with any PV in any gene are included in this table
b: Non-carrier group is composed of individuals without any detectable PV or VUS (negative result)
c: Carriers of PVs vs non-carriers. Wilcox test, two-sided, was used for continuous variables (age). Fisher test was used for categorical variables.

Table S4. Clinical and demographic characteristics of patients diagnosed with breast, colorectal, and ovarian cancer (continued)

Characteristic		All (n = 64)	Carriers of PVs[a] (n = 17)	Non-carriers[b] (n = 23)	p-value[c]	VUS (n = 24)
**Ovarian cancer**						
**Age at diagnosis (years)**	Median [IQR]	50.5 [13.2]	51.0 [6.00]	49.0 [21.0]	0.416	51.5 [13.5]
**Age at diagnosis (years), n (%)**	≤ 50	32 (50.0)	8 (47.1)	13 (56.5)	0.553	11 (45.8)
	> 50	32 (50.0)	9 (52.9)	10 (43.5)		13 (54.2)
**Histopathology, n (%)**	Carcinoma	1 (1.60)	0 (0.00)	1 (4.30)	0.097	0 (0.00)
	Clear cells	2 (3.10)	0 (0.00)	1 (4.30)		1 (4.20)
	Endometrioid	3 (4.70)	0 (0.00)	3 (13.0)		0 (0.00)
	Mucinous	4 (6.20)	0 (0.00)	3 (13.0)		1 (4.20)
	Serous	52 (81.2)	17 (100)	14 (60.9)		21 (87.5)
	Granulosa cells	1 (1.60)	0 (0.00)	1 (0.00)		0 (0.00)
	Missing data	1 (1.60)	0 (0.00)	0 (0.00)		1 (4.20)
**Tumor grade, n (%)**	High grade	40 (62.5)	14 (82.4)	13 (56.5)	0.600	13 (54.2)
	Low grade	10 (15.6)	1 (5.90)	3 (13.0)		6 (25.0)
	Missing data	14 (21.9)	2 (11.8)	7 (30.4)		5 (20.8)
**Locations, n (%)**	Unilateral	16 (25.0)	2 (11.8)	6 (26.1)	0.183	8 (33.3)
	Bilateral	18 (28.1)	9 (52.9)	5 (21.7)		4 (16.7)
	Missing data	30 (46.9)	6 (35.3)	12 (52.2)		12 (50.0)
**Stage, n (%)**	Early	14 (21.9)	5 (29.4)	7 (30.4)	1.00	2 (8.30)
	Locally advanced	29 (45.3)	7 (41.2)	8 (34.8)		14 (58.3)
	Metastatic	13 (20.3)	4 (23.5)	5 (21.7)		4 (16.7)
	Missing data	8 (12.5)	1 (5.90)	3 (13.0)		4 (16.7)

IQR: Interquartile range; PVs: Pathogenic/Likely Pathogenic variants; VUS: variant of uncertain significance

a: Patients with any PV in any gene are included in this table

b: Non-carrier group is composed of individuals without any detectable PV or VUS (negative result)

c: Carriers of PVs vs non-carriers. Wilcox test, two-sided, was used for continuous variables (age). Fisher test was used for categorical variables.

Table S5. Characteristics of patients diagnosed with other cancers

System Group (n = 99)	Genetic test result	n (%)	Tumor/cancer type (n)	Age at diagnosis (years)
Endocrine (n = 25)	Carriers of PVs[a]	6 (24.0)	Pituitary Adenoma (1)<br>Thyroid (5)	21.0<br>42.0[c]
	Non-carriers[b]	13 (52.0)	Adrenal cortex (1)<br>Thyroid (12)	42.0<br>46.0[c]
	VUS	6 (24.0)	Thyroid (6)	53.5[c]
Gastrointestinal (n = 8)[d]	Carriers of PVs[a]	2 (25.0)	Pancreatic (2)	31.5[c]
	Non-carriers[b]	4 (50.0)	Pancreatic (2)<br>Ampullary (1)<br>Small Intestine (1)	43.0[c]<br>46.0<br>41.0
	VUS	2 (25.0)	Pancreatic (1)<br>Gallbladder (1)	63.0<br>60.0
Gynecological (n = 9)[e]	Carriers of PVs[a]	1 (11.1)	Endometrial (1)	56.0
	Non-carriers[b]	3 (33.3)	Endometrial (3)	55.0[c]
	VUS	5 (50.0)	Endometrial (3)<br>Müllerian tumor (2)	50.0[c]<br>65.0[c]
Melanoma (n = 13)	Carriers of PVs[a]	2 (15.4)	Melanoma (2)	22.0[c]
	Non-carriers[b]	3 (23.1)	Melanoma (3)	44.0[c]
	VUS	8 (61.5)	Melanoma (8)	50.0[c]
Skin/Non-melanoma (n = 7)	Carriers of PVs[a]	0 (0.00)	-	-
	Non-carriers[b]	3 (42.8)	Other skin (2)<br>Squamous Cell (1)	38.5[c]<br>28.0
	VUS	4 (57.1)	Basal Cell (4)	48.5[c]
Respiratory (n = 4)	Carriers of PVs[a]	1 (25.0)	Lung (1)	57.0
	Non-carriers[b]	2 (50.0)	Lung (1)<br>Bronchial carcinoid (1)	68.0<br>45.0
	VUS	1 (25.0)	Lung (1)	33.0
Bones/Connective/Other soft tissues (n = 14)	Carriers of PVs[a]	0 (0.00)	-	-
	Non-carriers[b]	6 (40.0)	Sarcoma (6)	43.5[c]
	VUS	8 (60.0)	Sarcoma (8)	49.5[c]
Central/Peripheral Nervous (n = 5)	Carriers of PVs[a]	1 (20.0)	Schwannoma (1)	33.0
	Non-carriers[b]	3 (60.0)	Schwannoma (1)<br>Peripheral or CNS (2)	34.0<br>37.0[c]
	VUS	1 (20.0)	Schwannoma (1)	51.0
Urinary tract (n = 14)	Carriers of PVs[a]	3 (21.4)	Prostate (1)<br>Kidney (2)	82.0<br>39.5[c]
	Non-carriers[b]	5 (35.7)	Prostate (3)<br>Kidney (1)<br>Urothelial (1)	60.0[c]<br>53.0<br>52.0
	VUS	6 (42.8)	Kidney (5)<br>Prostate (1)	52.0[c]<br>65.0

PVs: Pathogenic/Likely Pathogenic variants; VUS: variant of uncertain significance
a: Patients with any PV in any gene are included in this table
b: Non-carrier group is composed of individuals without any detectable PV or VUS (negative result)
c: Median
d: Gastrointestinal cases category does not include colorectal cancers (analyzed in Supplemental Table S4). Gastric cancer cases are excluded from this analysis
e: Gynecological cases category does not include ovarian cancers (analyzed in Supplemental Table S4)

3. **Supplementary Figures:**

Figure S1. Place of birth reported by all the patients registered in the Hereditary Cancer Program at the INC-C (n = 986)

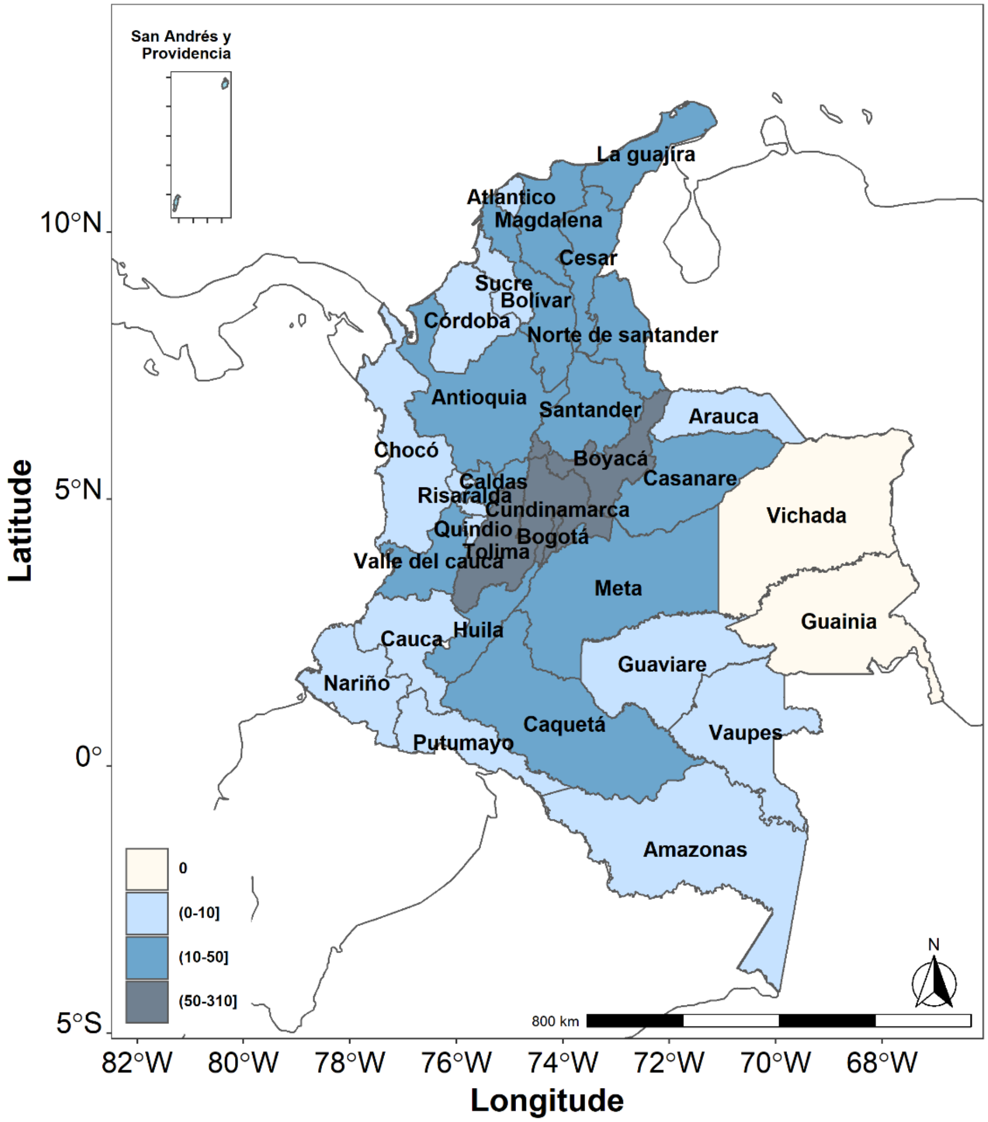

Geo-map showing the place of birth reported by all the patients registered in the REDCap database created for the Institutional Hereditary Cancer Program, from April 2018 to June 2020, across Colombian departments. Place of birth was recorded for 959 patients, and 27 had missing entries.

Figure S2. Domicile reported by all the patients registered in the Hereditary Cancer Program at the INC-C (n = 986)

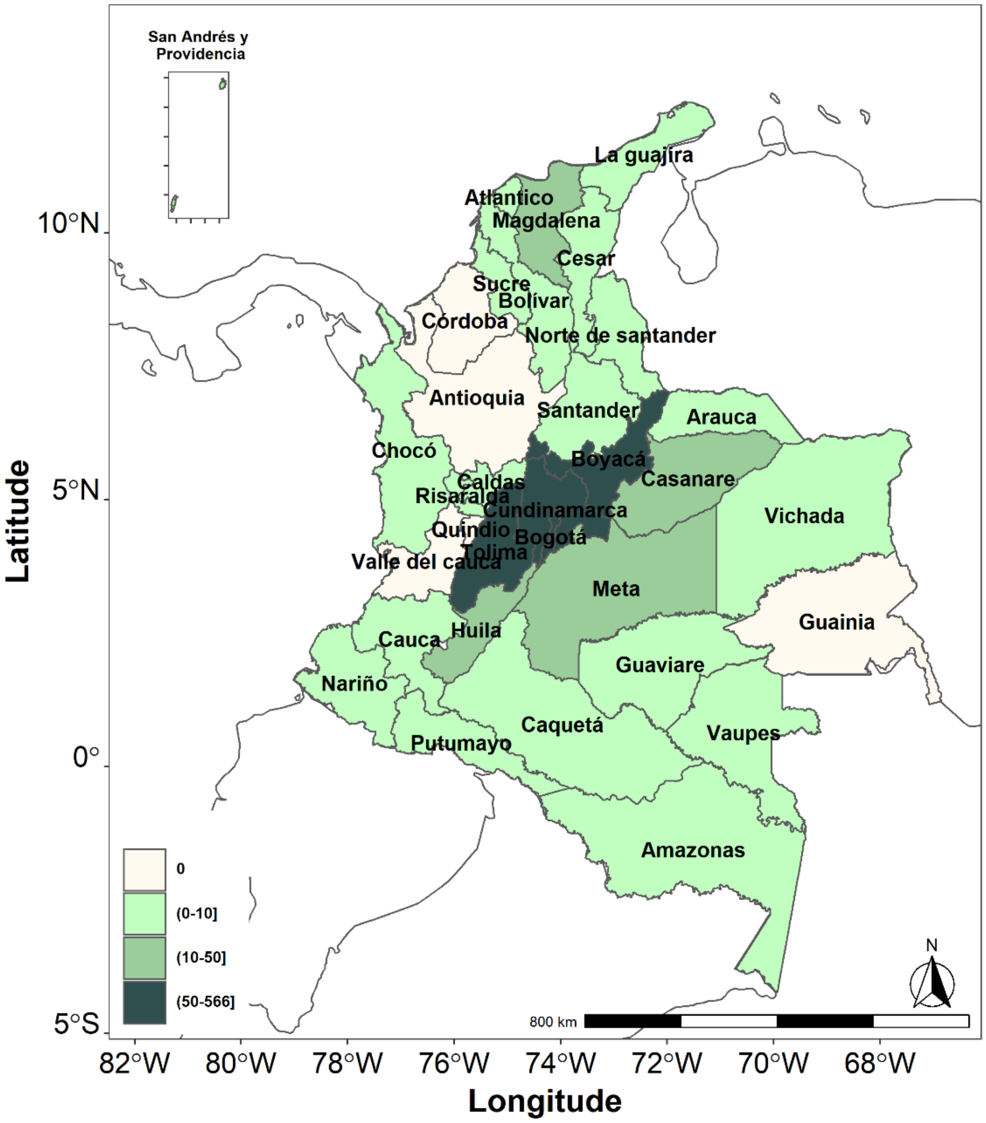

Geo-map showing the domicile reported by all the patients registered in the REDCap database created for the Institutional Hereditary Cancer Program, from April 2018 to June 2020, across Colombian departments. Domicile was recorded for all cases.

Figure S3. Age at diagnosis and sex distribution by cancer type of all the patients registered in the Hereditary Cancer Program at the INC-C (n = 986)

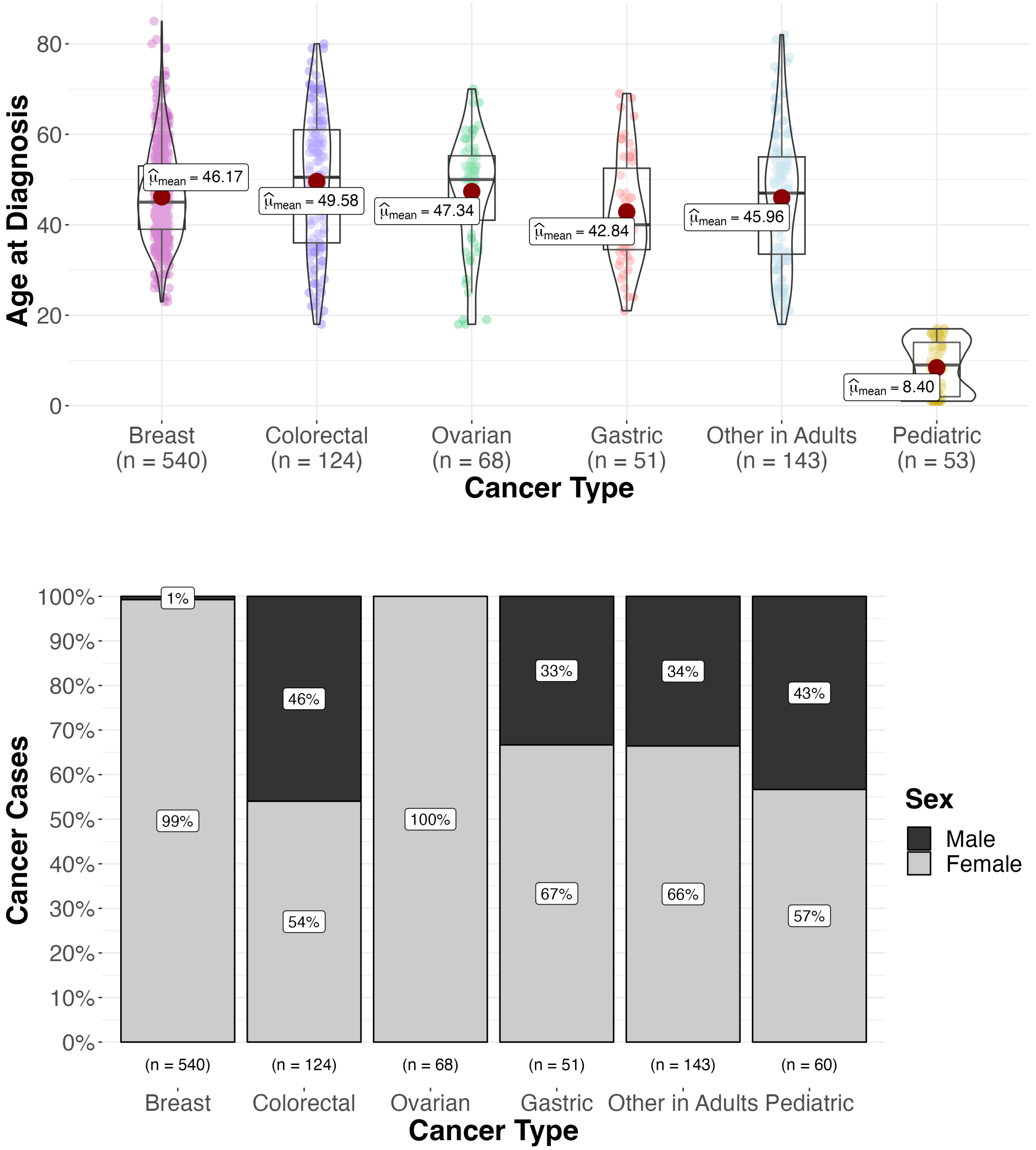

Mean age at diagnosis (Top Panel; Violin Plot) and sex proportions (Bottom Panel; Bar Plot) by cancer type of all the 986 patients registered in the REDCap database created for the Institutional Hereditary Cancer Program, from April 2018 to June 2020. For seven pediatric patients, the age at diagnosis was missing. Among referred cases, a total of 143 were categorized as other cancers in adults or “other in adults” group, but 17 cases were later excluded (i.e., 12 pheochromocytoma/paraganglioma cases, three hematological malignancies and two cases with unknown primary). After excluding these, 126 “other in adults” cases remained. Most breast (91%; 491/540), colorectal (93%; 115/124), ovarian (94%; 64/68), and other adult cancer (79%; 99/126) patients included in the program underwent genetic testing, while some patients did not meet the criteria or were not interested in testing. These cases with genetic testing results are further analyzed in this study (Supplemental Tables S4-S5).

Figure S4. Mismatch repair (MMR) immunohistochemistry (IHC) patterns and corresponding pathogenic/likely pathogenic variants (PVs) in patients with colorectal cancer (n = 115)

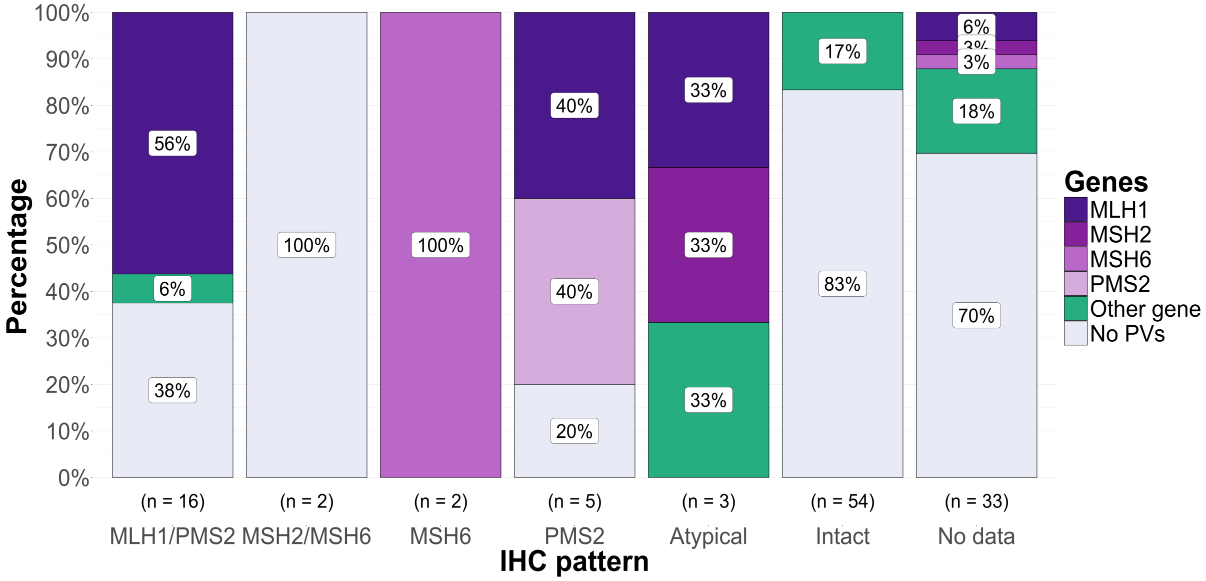

Stacked bar plot showing the percentage distribution of pathogenic/likely pathogenic variants (PVs) in mismatch repair (MMR) genes across different immunohistochemistry (IHC) patterns (loss of expression of MLH1/PMS2, MSH2/MSH6, isolated MSH6, isolated PMS2, Atypical, Intact, or No data) in patients with colorectal cancer. Atypical: includes cases with MLH1/PMS2/MSH6 (n = 2) or MSH2/MSH6/PMS2 (n = 1) loss of expression. Intact: represents normal MMR protein staining. No data: accounts for missing IHC results. Other gene: refers to non-MMR genes. No PVs: indicates cases where no PVs were detected.

Figure S5. Prevalence of hereditary cancer syndromes (HCS) calculated for adult cancer cases at risk and with genetic test results (n = 769)

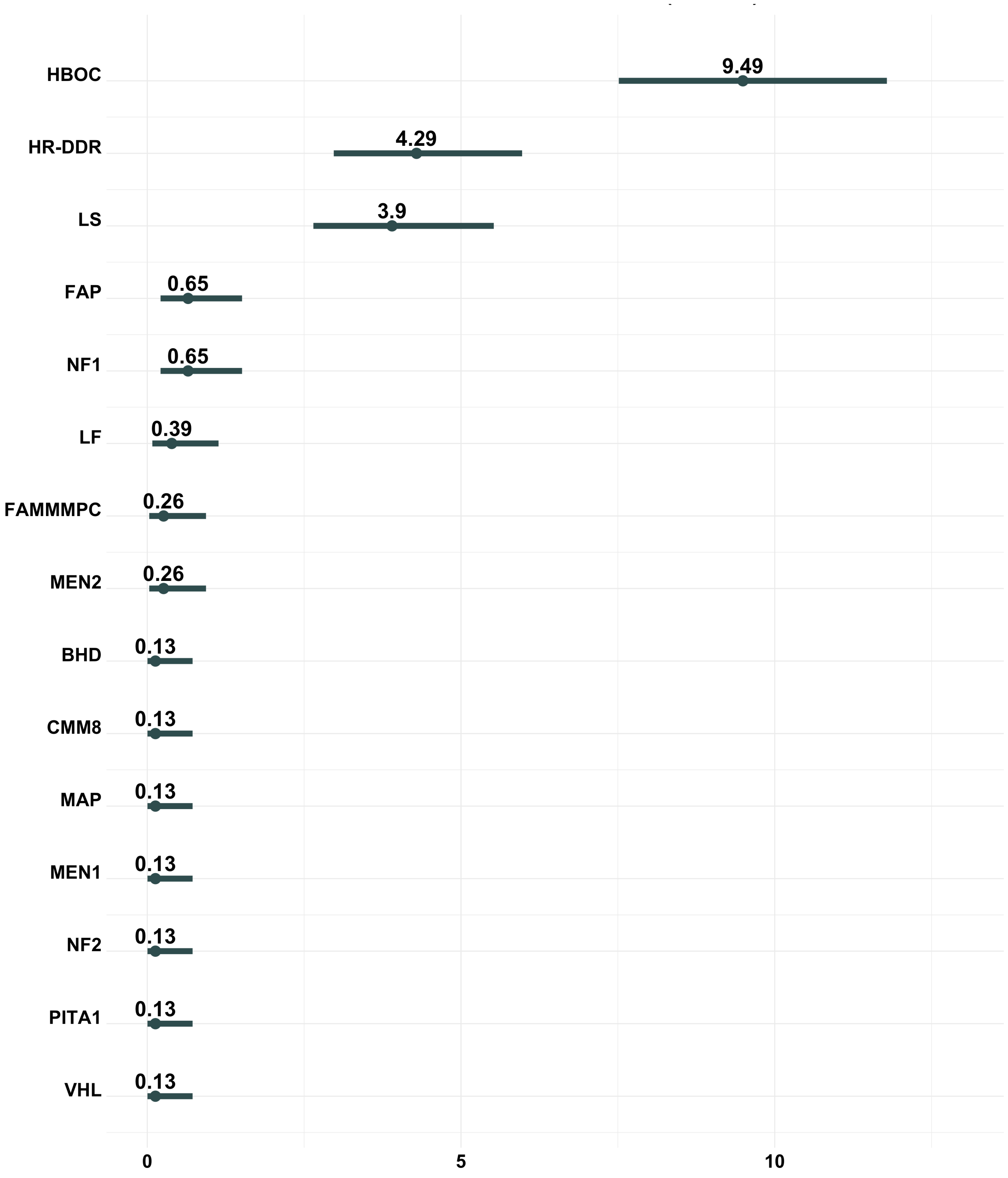

Ranked error bar plot showing the overall prevalence calculation for each hereditary cancer syndrome (HCS), and its 95% confidence interval, per 100 adult cancer cases at risk with genetic test results (n = 769), that are registered in the REDCap database created for the Institutional Hereditary Cancer Program, from April 2018 to June 2020. This graphic includes findings in genes that are known to be associated with the patient's cancer type and those in genes that have not been associated with the patient's current disease (i.e., incidental findings). HBOC, hereditary breast and ovarian cancer *BRCA1* and *BRCA2* related; HR-DDR, homologous recombination DNA damage repair (i.e., *ATM*, *BARD1*, *BRIP1*, *CHEK2*, *PALB2*, *RAD51C*, and *RAD51D*) associated cancer risk; LS, Lynch syndrome (i.e, *MLH1*, *PMS2*, *MSH6,*

and *MSH2*); FAP, familial adenomatous polyposis (i.e., *APC*); NF1, neurofibromatosis type 1 (i.e., *NF1*); LF, Li-Fraumeni syndrome (i.e., *TP53*); FAMMMPC, familial atypical multiple mole melanoma-pancreatic carcinoma syndrome (i.e., *CDKN2A*); MEN2, multiple endocrine neoplasia type 2 (i.e., *RET*); BHD, Birt-Hogg Dube syndrome (i.e., *FLCN*); CMM8, cutaneous malignant melanoma-8 syndrome (i.e., *MITF*); MAP, MUTYH-associated polyposis (i.e., biallelic *MUTYH*); MEN1, multiple endocrine neoplasia type 1 (i.e., *MEN1*); NF2, neurofibromatosis type 2 (i.e., *NF2*); PITA1, pituitary adenoma 1 predisposition (i.e., *AIP*); VHL, Von Hippel-Lindau syndrome (i.e., *VHL*).

Figure S6. Distribution and effect of pathogenic/likely pathogenic variants (PVs) in *BRCA1* and *BRCA2* genes

A

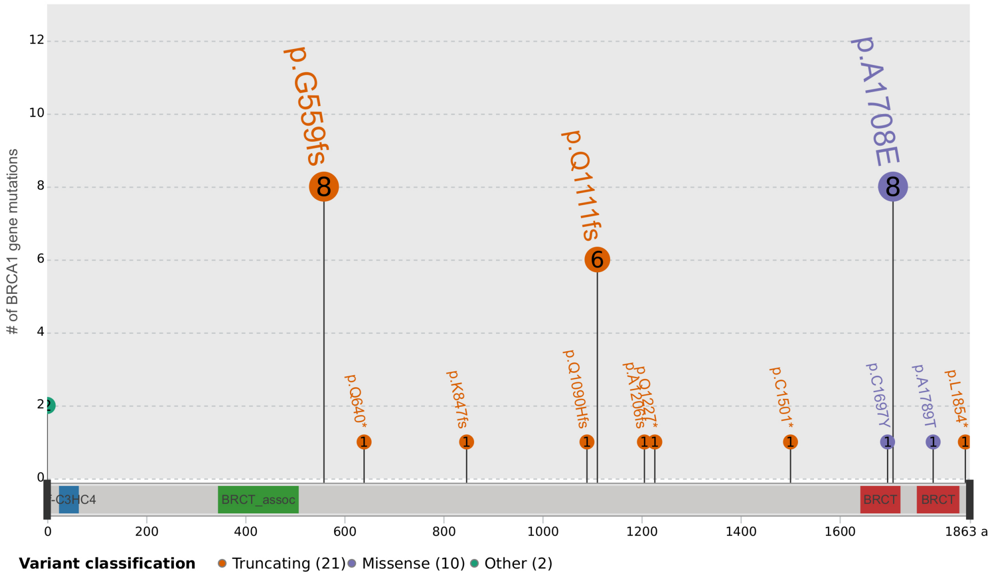

B

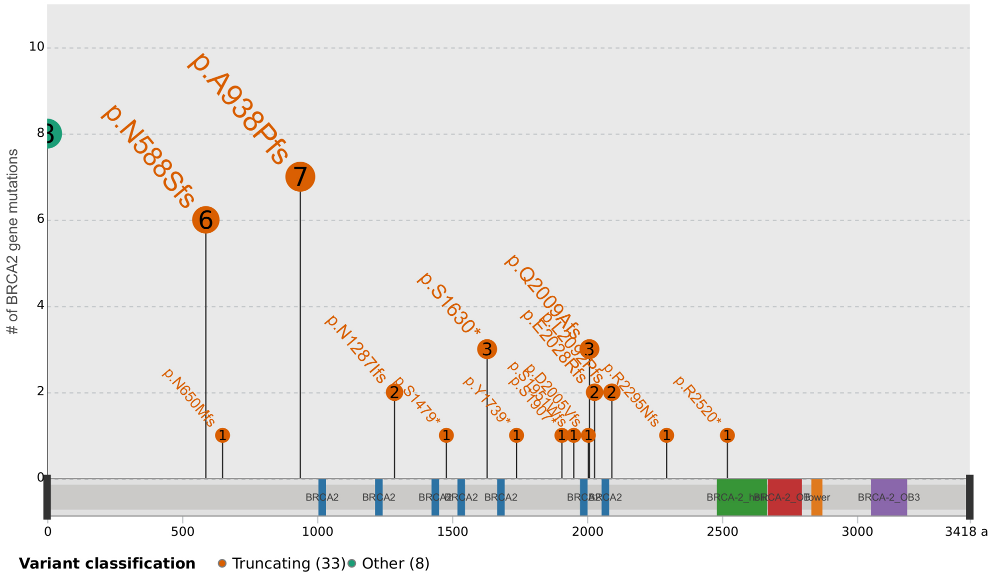

G3viz lollipop-diagrams showing graphical representations of germline pathogenic/likely pathogenic variants (PVs) detected in *BRCA1* (A) and *BRCA2* (B) genes. Both plots show the distribution and effect of these PVs per gene in our high-risk Colombian cohort. The Pfam protein domains63 and the positions of specific PVs are shown (x-axis), along with the number of carriers (y-axis). PVs different from truncating (color red) or missense (color purple) are grouped as “other” (color green). Other PVs in *BRCA1* (found in two carriers) are located in the position 0.1 in the x-axis, and these correspond to two different intronic variants that affect RNA splicing and likely results in an absent or truncated protein product [*BRCA1*

c.212+1G>T and c.213-12A>G]. Other PVs in *BRCA2* (found in eight carriers) are also located in the position 0.1 in the x-axis; these alterations can cause absent or non-functional protein product, one is an intronic variant [*BRCA2* c.632-3C>G] detected in two carriers and the rest correspond to two different CNVs detected in five [*BRCA2* ex1-14del] and in one case [*BRCA2* ex12-16del].

Well-known recurrent PVs in *BRCA1* are: [c.1674del p.(Gly559ValfsTer13), c.3331_3334del p.(Gln1111AsnfsTer5), and c.5123C>A p.(Ala1708Glu)] [6-9]. Well-known recurrent PVs in *BRCA2* are: [c.1763_1766del p.(Asn588SerfsTer25); c.6024dup p.(Gln2009AlafsTer9); c.4889C>G p.(Ser1630Ter)] [7, 9, 10]. The CNV *BRCA2* ex1-14del, has recently been reported in two Colombian families that shared a conserved haplotype implying that it may have arisen from a common founder [10].

References

1. Manotas MC, Rivera AL, Sanabria-Salas MC. Variant curation and interpretation in hereditary cancer genes: An institutional experience in Latin America. Mol Genet Genomic Med. 2023; https://doi.org/doi:10.1002/mgg3.2141.

2. Manotas MC, Rivera AL, Gomez AM, Abisambra P, Guevara G, Medina V, et al. SDHB exon 1 deletion: A recurrent germline mutation in Colombian patients with pheochromocytomas and paragangliomas. Front Genet. 2022; https://doi.org/doi:10.3389/fgene.2022.999329.

3. Richards S, Aziz N, Bale S, Bick D, Das S, Gastier-Foster J, et al. Standards and guidelines for the interpretation of sequence variants: a joint consensus recommendation of the American College of Medical Genetics and Genomics and the Association for Molecular Pathology. Genet Med. 2015; https://doi.org/doi:10.1038/gim.2015.30.

4. Freeman PJ, Hart RK, Gretton LJ, Brookes AJ, Dalgleish R. VariantValidator: Accurate validation, mapping, and formatting of sequence variation descriptions. Hum Mutat. 2018; https://doi.org/doi:10.1002/humu.23348.

5. Sirugo G, Williams SM, Tishkoff SA. The Missing Diversity in Human Genetic Studies. Cell. 2019; https://doi.org/doi:10.1016/j.cell.2019.02.048.

6. Blay P, Santamaria I, Pitiot AS, Luque M, Alvarado MG, Lastra A, et al. Mutational analysis of BRCA1 and BRCA2 in hereditary breast and ovarian cancer families from Asturias (Northern Spain). BMC Cancer. 2013; https://doi.org/doi:10.1186/1471-2407-13-243.

7. Dutil J, Golubeva VA, Pacheco-Torres AL, Diaz-Zabala HJ, Matta JL, Monteiro AN. The spectrum of BRCA1 and BRCA2 alleles in Latin America and the Caribbean: a clinical perspective. Breast Cancer Res Treat. 2015; https://doi.org/doi:10.1007/s10549-015-3629-3.

8. Herzog JS, Chavarri-Guerra Y, Castillo D, Abugattas J, Villarreal-Garza C, Sand S, et al. Genetic epidemiology of BRCA1- and BRCA2-associated cancer across Latin America. NPJ Breast Cancer. 2021; https://doi.org/doi:10.1038/s41523-021-00317-6.

9. Ossa Gomez CA, Achatz MI, Hurtado M, Sanabria-Salas MC, Sullcahuaman Y, Chavarri-Guerra Y, et al. Germline Pathogenic Variant Prevalence Among Latin American and US Hispanic Individuals Undergoing Testing for Hereditary Breast and Ovarian Cancer: A Cross-Sectional Study. JCO Glob Oncol. 2022; https://doi.org/doi:10.1200/GO.22.00104.

10. Torres D, Bermejo JL, Rashid MU, Briceno I, Gil F, Beltran A, et al. Prevalence and Penetrance of BRCA1 and BRCA2 Germline Mutations in Colombian Breast Cancer Patients. Sci Rep. 2017; https://doi.org/doi:10.1038/s41598-017-05056-y.
